# Not out of the Mediterranean: Atlantic populations of the gorgonian *Paramuricea clavata* are a separate sister species under further lineage diversification

**DOI:** 10.1002/ece3.9740

**Published:** 2023-01-29

**Authors:** Márcio A. G. Coelho, Gareth A. Pearson, Joana R. H. Boavida, Diogo Paulo, Didier Aurelle, Sophie Arnaud‐Haond, Daniel Gómez‐Gras, Nathaniel Bensoussan, Paula López‐Sendino, Carlo Cerrano, Silvija Kipson, Tatjana Bakran‐Petricioli, Eliana Ferretti, Cristina Linares, Joaquim Garrabou, Ester A. Serrão, Jean‐Baptiste Ledoux

**Affiliations:** ^1^ Centre for Marine Sciences (CCMAR) University of Algarve Faro Portugal; ^2^ MARE – Marine and Environmental Sciences Centre ISPA‐Instituto Universitário Lisboa Portugal; ^3^ Aix Marseille Univ., Université de Toulon, CNRS, IRD, MIO Marseille France; ^4^ Institut de Systématique, Evolution, Biodiversité (ISYEB), Muséum National d'Histoire Naturelle, CNRS Sorbonne Université Paris France; ^5^ MARBEC (Marine Biodiversity, Exploitation and Conservation) Univ. Montpellier, IFREMER, CNRS, IRD Sète Cedex France; ^6^ Hawai‘i Institute of Marine Biology University of Hawai‘i at Mānoa Kaneohe Hawaii USA; ^7^ Departament de Biologia Evolutiva, Ecologia i Ciències Ambientals Universitat de Barcelona (UB) Barcelona Spain; ^8^ Institut de Recerca de la Biodiversitat (IRBio) Universitat de Barcelona (UB) Barcelona Spain; ^9^ Departament de Biologia Marina Institut de Ciències del Mar (CSIC) Barcelona Spain; ^10^ Dipartimento di Scienze della Vita e dell’Ambiente (DiSVA) Università Politecnica delle Marche Ancona Italy; ^11^ Consorzio Nazionale Interuniversitario per le Scienze del Mare (CoNISMa) Rome Italy; ^12^ Stazione Zoologica Anton Dohrn Naples Italy; ^13^ Fano Marine Center Fano Italy; ^14^ Department of Biology, Faculty of Science University of Zagreb Zagreb Croatia; ^15^ SEAFAN – Marine Research & Consultancy Zagreb Croatia; ^16^ Studio Associato GAIA s.n.c. Genoa Italy; ^17^ Institute of Marine Science The University of Auckland Auckland New Zealand; ^18^ CIBIO/InBIO‐Centro de Investigação em Biodiversidade e Recursos Genéticos Vairão Portugal; ^19^ CIIMAR/CIMAR, Centro Interdisciplinar de Investigação Marinha e Ambiental Universidade do Porto Porto Portugal

**Keywords:** Atlantic‐Mediterranean transition, cryptic diversity, incomplete lineage sorting, Octocorallia, phylotranscriptomics, species delimitation

## Abstract

The accurate delimitation of species boundaries in nonbilaterian marine taxa is notoriously difficult, with consequences for many studies in ecology and evolution. Anthozoans are a diverse group of key structural organisms worldwide, but the lack of reliable morphological characters and informative genetic markers hampers our ability to understand species diversification. We investigated population differentiation and species limits in Atlantic (Iberian Peninsula) and Mediterranean lineages of the octocoral genus *Paramuricea* previously identified as *P. clavata*. We used a diverse set of molecular markers (microsatellites, RNA‐seq derived single‐copy orthologues [SCO] and *mt‐mutS* [mitochondrial barcode]) at 49 locations. Clear segregation of Atlantic and Mediterranean lineages was found with all markers. Species‐tree estimations based on SCO strongly supported these two clades as distinct, recently diverged sister species with incomplete lineage sorting, *P.* cf. *grayi* and *P. clavata*, respectively. Furthermore, a second putative (or ongoing) speciation event was detected in the Atlantic between two *P.* cf. *grayi* color morphotypes (yellow and purple) using SCO and supported by microsatellites. While segregating *P.* cf. *grayi* lineages showed considerable geographic structure, dominating circalittoral communities in southern (yellow) and western (purple) Portugal, their occurrence in sympatry at some localities suggests a degree of reproductive isolation. Overall, our results show that previous molecular and morphological studies have underestimated species diversity in *Paramuricea* occurring in the Iberian Peninsula, which has important implications for conservation planning. Finally, our findings validate the usefulness of phylotranscriptomics for resolving evolutionary relationships in octocorals.

## Graphical abstract

Demarcating the transition between intraspecies lineages and speciation requires accurate delimitation of species boundaries, which in many marine invertebrates such as corals is notoriously difficult, leading to misinterpretations of their ecology and evolution. This study investigates species boundaries, taxonomic affinity, and lineage diversification for key structural species of branching octocorals in the genus *Paramuricea* across the Atlantic‐Mediterranean biogeographic regions. We combine species tree methods (using RNA‐seq data) with population genetics (clustering methods using microsatellites) and phylogenetics (mitochondrial barcode) to show that the Atlantic and Mediterranean clades studied are recently diverged sister species on separate evolutionary trajectories (*Paramuricea* cf. *grayi* and *P. clavata*), also revealing a second putative (or ongoing) speciation event within Atlantic *P.* cf. *grayi*.

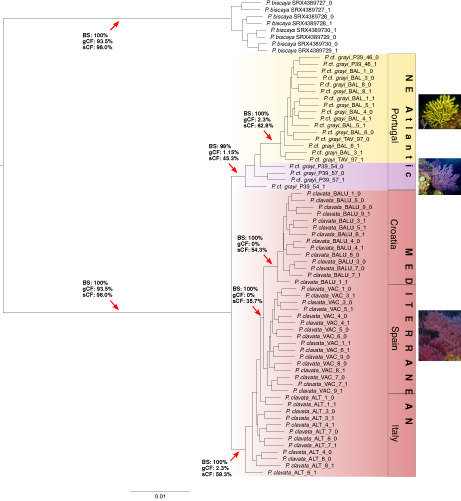

## 1 INTRODUCTION

The species is the fundamental unit of analysis in ecology and evolution, from field and behavioral ecology to population genetics, phylogeography, and systematics (De Queiroz, 2005). Thus, species delimitation sets the tone for research questions in a myriad of fields, with critical implications for biodiversity assessment and conservation planning (Hey et al., 2003; Pante et al., 2015; Sukumaran & Knowles, 2017). However, identifying species boundaries is challenging in many taxa for several reasons, including phenotypic plasticity (Pica et al., 2018; Pino‐Bodas et al., 2011; Sánchez et al., 2007), lack of diagnostic morphological characters (Erpenbeck et al., 2006; McFadden et al., 2017), homoplasy (Baker et al., 2000; Morrow et al., 2013; Waeschenbach et al., 2009), low‐resolution of DNA barcoding markers (Calderón et al., 2006; Huang et al., 2008; Shearer et al., 2002), incomplete lineage sorting (ILS) in recently diverged taxa (Aurelle et al., 2017; Meyer et al., 2017; Pollard et al., 2006), and introgressive hybridization (Combosch & Vollmer, 2015; Padilla‐García et al., 2018; reviewed in Harrison & Larson, 2014). The latter phenomenon is generally the result of complex biogeographic histories in which populations that diverged in allopatry come into secondary contact. Identifying barriers to gene flow and understanding how genetic differences accumulate over time is an inherently challenging task, particularly for marine systems in which these barriers are less obvious than for their terrestrial counterparts.

The Atlantic‐Mediterranean transition provides an interesting geographic setting to study population differentiation and speciation in the marine realm (Borsa et al., 1997; Patarnello et al., 2007). This narrow region has a complex biogeographical history, involving dramatic geological events that affected the entire Mediterranean Sea, including the Messinian Salinity Crisis and subsequent Zanclean flood (5.96 and 5.33 Ma ago, respectively; Garcia‐Castellanos et al., 2009; Garcia‐Castellanos & Villaseñor, 2011; Krijgsman et al., 1999), as well as sea level fluctuations during glacial cycles (Vacchi et al., 2016). These, in turn, have contributed to multiple episodes of species extinction‐recolonization or allopatry‐secondary contact between the Atlantic and Mediterranean basins, which have shaped contemporary patterns of genetic differentiation across this range (Alberto et al., 2008; Duranton et al., 2018; reviewed in Patarnello et al., 2007). Within the Mediterranean, several oceanographic discontinuities exist, among which is the Almeria‐Oran Front (AOF), a large‐scale density front at the eastern edge of the East Alboran gyre that extends southwards from Almeria (SE Spain) to Oran (Algeria) (Millot, 1999; Tintore et al., 1988). The AOF constitutes a barrier to gene flow in many species, often resulting in complex phylogeographical patterns (Patarnello et al., 2007), particularly in taxa with restricted dispersal such as seagrasses (Alberto et al., 2008), sponges (Dailianis et al., 2011; Riesgo et al., 2019), and corals (Casado‐Amezúa et al., 2012; Mokhtar‐Jamaï et al., 2011). Indeed, genetic discontinuities associated with the AOF are even reported for species with high dispersal potential such as the European sea bass (*Dicentrarchus labrax*; Duranton et al., 2018; Lemaire et al., 2005; Naciri et al., 1999; Tine et al., 2014).

Branching octocorals of the genus *Paramuricea* (Octocorallia: Paramuriceidae) are important constituents of many shallow and deep‐sea communities of benthic suspension feeders (e.g., corals, sponges, and bivalves) in the Atlantic Ocean and Mediterranean Sea, often forming extensive, dense assemblages supporting high levels of biodiversity (Carpine & Grasshoff, 1975; Cerrano et al., 2010; Doughty et al., 2014; Grasshoff, 1977; Grinyó et al., 2016; Nestorowicz et al., 2021; Ponti et al., 2018; Walting et al., 2011). Currently, seven valid species are known to occur along the NE Atlantic and Mediterranean (Carpine & Grasshoff, 1975; Grasshoff, 1977; WoRMS, 2022), with a further two putatively new species recently discovered after re‐examination of natural history collections (Sampaio et al., 2019). Two of these, *Paramuricea clavata* and *Paramuricea macrospina*, were historically regarded as endemic to the Mediterranean Sea (Carpine & Grasshoff, 1975; Grasshoff, 1977; Pica et al., 2018) despite several recent studies extending the distribution ranges of *P. clavata* to adjacent Atlantic coastlines (Boavida, Assis, et al., 2016; Cúrdia et al., 2012; Pilczynska et al., 2019; Pilczynska, Cocito, et al., 2017) and of *P. macrospina* potentially to the Cape Verde archipelago (Sampaio et al., 2019).

The red gorgonian *P. clavata* is a major structuring species of Mediterranean coralligenous assemblages, generally restricted to low‐light (Ballesteros, 2006), reaching densities well above 20 colonies m^−2^ (Gori et al., 2011; Linares, Coma, Garrabou, et al., 2008; Ponti et al., 2018). The genetic structure of *P. clavata* in the Mediterranean shows sharp regional genetic breaks, strong differentiation at 10–100 s of meters and significant isolation by distance (IBD) at both regional and local scales (Arizmendi‐Mejía et al., 2015; Ledoux et al., 2018; Mokhtar‐Jamaï et al., 2011, 2013). Spatial genetic structure in *P. clavata* is congruent with oceanographic barriers to gene flow (AOF: Mokhtar‐Jamaï et al., 2011; seasonal cyclonic gyres along the Adriatic: Ledoux et al., 2018), and with the limited dispersal ability inherent to a reproductive strategy of larval brooding on maternal colonies (as observed in other brooding corals: Ayre & Hughes, 2000; Ledoux, Mokhtar‐Jamaï, et al., 2010; Mokhtar‐Jamaï et al., 2013; Smilansky & Lasker, 2014; Underwood et al., 2007). Atlantic populations identified as *P. clavata* along the Portuguese coast are genetically differentiated from Mediterranean ones and also differentiated between western and southern Portugal (Pilczynska et al., 2019; Pilczynska, Cocito, et al., 2017). Interestingly, this differentiation between western and southern populations in Portugal appears to coincide with color morphotypes that dominate circalittoral coral gardens in each region (Pilczynska, Cocito, et al., 2017), with a yellow morphotype prevalent in the Algarve (southern Portugal) and a purple morphotype dominating communities in western Portugal. Although both morphotypes can occur in sympatry, our field surveys indicate that the purple morphotype is extremely rare in the south (Dias et al., 2020; colonies reported as *P. clavata*) and vice‐versa (J. Boavida and M. Coelho, personal observations). Despite these findings, the existence and exact location of the Atlantic‐Mediterranean break, and the level of divergence across these regions (i.e., whether it represents within‐species population structure or speciation processes) remains unknown in *P. clavata* due to incomplete geographic sampling and low marker resolution (Mokhtar‐Jamaï et al., 2011; Pilczynska et al., 2019; Pilczynska, Cocito, et al., 2017). Indeed, standard DNA barcoding approaches for species delimitation are poorly suited to resolve closely related or recently diverged octocorals due to slow rates of mitochondrial gene evolution (Calderón et al., 2006; Hellberg, 2006; Huang et al., 2008; McFadden et al., 2011; Shearer et al., 2002). These issues obscure the relationship between Mediterranean and Atlantic populations. For instance, Mediterranean populations of the threatened precious red coral *Corallium rubrum* typically exhibit strong genetic differentiation at small spatial scales (Costantini et al., 2007; Ledoux, Garrabou, et al., 2010; Ledoux, Mokhtar‐Jamaï, et al., 2010). However, the Atlantic‐Mediterranean genetic break associated with the AOF is apparently lacking, presumably due to the extended influence of Atlantic populations in the Mediterranean gene pool, such as those recently rediscovered in SW Portugal (Aurelle et al., 2011; Boavida, Paulo, et al., 2016).

In addition to limited geographic sampling and marker resolution, the evolutionary relationships between *P. clavata* populations occurring across the Atlantic‐Mediterranean transition have also been obscured by the potential occurrence in sympatry of congeneric species with intergrading morphologies that are difficult to discriminate. For instance, the southern coast of Portugal is part of the distribution range of *Paramuricea grayi*, a species of deep littoral habitats historically described from the Lusitanian‐Mauritanian East Atlantic to the Gulf of Guinea (Grasshoff, 1977, 1992), but which has subsequently been described from Galicia and the Bay of Biscay (northern Iberian Peninsula) (Poliseno et al., 2017), among other locations in the NW Atlantic (Thoma, 2013; Walting et al., 2011). Importantly, recent phylogenetic reconstructions based on a mitochondrial marker and RAD‐sequencing (RAD‐seq) loci have shown that *P. grayi* is a sister species of *P. clavata* (Poliseno et al., 2017; Quattrini et al., 2022). The divergence between these two species could be either the result of a vicariance event in an Atlantic‐Mediterranean ancestor (Poliseno et al., 2017); or of divergence within the Mediterranean in which *P. clavata* diversified in mesophotic habitats from an ancestor with a broad depth range (Quattrini et al., 2022). Thus, it is possible that the Iberian Peninsula and adjacent areas form a contact zone between two recently diverged sister species (*P. clavata* in the Mediterranean and *P. grayi* in the NE Atlantic). Alternatively, the Atlantic populations of *P. clavata* reported from Portugal (Boavida, Assis, et al., 2016; Cúrdia et al., 2012; Pilczynska et al., 2019; Pilczynska, Cocito, et al., 2017) could be *P. grayi* misidentified as *P. clavata*. This highlights the need for further study of *Paramuricea* (and octocorals in general) along the Atlantic‐Mediterranean transition to better understand the evolutionary forces governing population differentiation and speciation in this genus across these important biogeographic regions. The delimitation of spatially significant and unique genetic units within a species is also necessary for emerging conservation policies aiming to preserve intraspecific genetic diversity and the associated adaptive potential. *Paramuricea clavata* needs such efforts given its key structuring role in ecosystem functioning across the Mediterranean (Rossi et al., 2017; Rossi & Rizzo, 2021), particularly with the growing recognition of its vulnerability to human disturbances and environmental changes (Cerrano et al., 2000; Garrabou et al., 2009, 2021; Linares et al., 2005; Linares, Coma, & Zabala, 2008). Resolving the nature of the divergence (i.e., population‐ or species‐level) across the Atlantic and Mediterranean is a necessary first step.

The aim of this study was to test genetic boundaries and species affinities within the genus *Paramuricea* across the Atlantic‐Mediterranean transition zone and assess the genetic diversity of the different lineages identified in these biogeographic regions. For this aim, the geographic scope includes the western and southern coasts of Portugal (Northeast Atlantic) and a large part of the distribution of *P. clavata* in the Mediterranean Sea, including the Adriatic and Aegean seas. We combined evidence from three types of molecular markers: (1) single‐copy orthologs (obtained from RNA‐seq data) for species delimitation and screening for cryptic species diversity; (2) microsatellite loci to analyze spatial genetic diversity and structure both across and within the Atlantic and Mediterranean regions considering alternatively lineage and population levels; and (3) the mitochondrial gene *mt‐mutS* (assembled from RNA‐seq read data) to extend taxonomic coverage of our phylogenetic analysis for the genus. We present evidence showing that previous assignments of Atlantic populations to *P. clavata* are incongruent with genomic data, and the taxa present along those coasts should instead be regarded as the morphospecies *P*. cf. *grayi*.

## 2 MATERIALS AND METHODS

### 2.1 Phylogenomic inference and species delimitation using RNA‐seq data

#### 2.1.1 Sample collection

**FIGURE 1:**
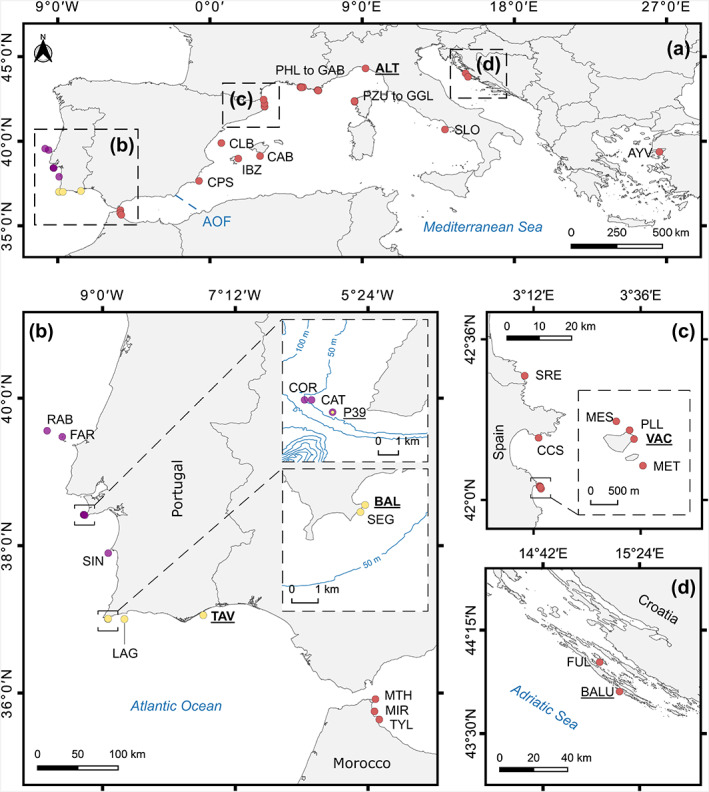
Map showing the sampling locations of *Paramuricea* across the NE Atlantic Ocean and Mediterranean Sea (a). Site codes follow the abbreviations of Table S1. Sites sampled for RNA‐seq are underlined, with populations that were also sampled for microsatellite genotyping shown in bold. PHL to GAB – all sites between Pharillons in the south of Marseille and Gabiniere in port‐Cros (France) as listed in Table S1; PZU to GGL – All sites in northern Corsica (France). For a detailed visualization of the location of Mediterranean sites see figure of Mokhtar‐Jamaï et al. (2011). AOF – approximate location of the Almeria‐Orán oceanographic front. (b) Detail of sampling sites along southern Iberia (NE Atlantic) and Alboran Sea (Mediterranean) separated by the strait of Gibraltar; insets show sites sampled in cape Espichel (upper) and Sagres (lower) with bathymetric isobaths shown as follows: 50 m, 100–500 m (increments of 100 m) and >500 m (increments of 200 m). (c) Locations sampled in Banyuls‐Sur‐Mer (southern France) and North‐Eastern Spain, including the Medes Islands. (d) Locations sampled along the eastern Adriatic Sea (Croatia). Colors of the markers correspond to species identified by SODA analysis (see results). Note that both the purple and yellow morphotypes of the *P*. cf. *grayi* were sampled at P39 (cape Espichel, western Portugal).

Gorgonian samples used for the phylogenomic analyses were subsampled from colonies used as controls in thermo‐tolerance experiments by Gómez‐Gras et al. (2022), focusing on four populations: Balun (Kornati, Croatia), Altare (Portofino, Italy), and La Vaca (Medes, Catalonia) for *P. clavata*; and Baleeira (Sagres, Portugal) for *P*. cf. *grayi* (Figure 1 and Table S1). Tissue from each of 6–8 individuals per population was sampled for RNA‐seq analysis (Table S1). The samples were preserved in RNAlater and stored at −80°C until further processing. In addition, four samples of *P*. cf. *grayi* were collected from two locations in southern (Tavira) and western (P39, Cape Espichel) Portugal. This included two specimens of the yellow color morph (1 from Tavira and 1 from P39) and two of the purple color morph from P39 (Table S1). These samples were flash frozen with liquid nitrogen upon collection and stored at −80°C until further processing.

### 2.2 RNA extraction and sequencing

Total RNA was extracted from a 0.5 to 1‐cm‐long branchlet piece. We coupled the TRIzol™ Plus RNA Purification Kit (Invitrogen) or the NZYol reagent (Nzytech) for tissue homogenization with the RNeasy Mini Kit (Qiagen) for RNA purification following the manufacturer's instructions. Residual DNA was removed using TurboDNAse (Invitrogen) or DNAse I (Qiagen). The quality, purity, and yield of the RNA was assessed using gel electrophoresis and a Qubit® Fluorometer and/or Nanodrop spectrophotometer. RNA‐seq library preparation and sequencing was outsourced to BGI Tech Solutions in Hong Kong (100 bp paired‐end DNBseq). A total of 34 individuals were processed for RNA‐seq and the sequences deposited in NCBI's Sequence Read Archive (BioProject ID: PRJNA847883; BioSample accessions SAMN28899278‐SAMN28899331).

### 2.3 Sequence quality control and filtering

Raw reads were preprocessed by BGI to trim adaptor sequences and low‐quality reads (<Q20) using filter_fq (https://github.com/greatfireball/filter_fq). The quality of filtered sequence data was then evaluated with two complementary quality control tools, FastQC v0.11.6 (https://www.bioinformatics.babraham.ac.uk/projects/fastqc/) and FastqPuri (Pérez‐Rubio et al., 2019). Because these analyses detected the presence of overrepresented sequences corresponding to ribosomal RNAs (rRNAs), contaminant rRNA reads were filtered with FastqPuri using the bloom filter approach implemented in the function trimFilterPE (for details see Pérez‐Rúbio et al., 2019 and the FastqPuri documentation page: https://github.com/jengelmann/FastqPuri). The input bloom filter was constructed from nuclear rRNA sequences downloaded from NCBI GenBank for the top octocoral blastn hit of the contaminants, which corresponded to the octocoral *Dendronephthya gigantea*.

### 2.4 De novo transcriptome assembly and completeness

Reference transcriptomes were assembled de novo with rnaSPAdes using default parameters (Bushmanova et al., 2019). In total, we performed four independent transcriptome assemblies using the sequence data of all samples from each population, one for samples from Baleeira (*P*. cf. *grayi*) and three for samples from Balun, Altare and La Vaca (*P. clavata*). The assemblies were subjected to multiple steps of quality control and curation. First, the quality of each assembly was evaluated with TransRate v1.0.1 using both sequence‐based and read mapping (onto the assembled contigs) metrics (Smith‐Unna et al., 2016). The read‐metrics mode measures the quality of the assembled contigs based on evidence from the reads used to generate the assembly, allowing the filtration of “bad” (i.e., poorly assembled) contigs from the assembly (Smith‐Unna et al., 2016). Second, using the “good” contigs obtained with TransRate, we identified putative open reading frames (ORFs) with FragGeneScan (Rho et al., 2010) and clustered the ORFs at 97% nucleotide sequence identity using VSEARCH (Rognes et al., 2016). Finally, the contigs of the clustered assemblies were aligned against NCBI's nonredundant database for Anthozoa (Taxonomy ID: 6101) using DIAMOND in BLASTX mode (Buchfink et al., 2014). Contigs with a top query hit were retained in the final transcriptome assemblies, with the remainder discarded as potential contaminants (e.g., bacteria).

The completeness of the final, curated transcriptome assemblies was evaluated with BUSCO v2/v3 by screening the recovery of single‐copy orthologs (queried as predicted proteins) that are expected to be present across higher taxonomic groups (Simão et al., 2015). The analysis was performed against the Metazoa reference database (OrthoDB 9) using the online platform gVolante v1.2.1 available at https://gvolante.riken.jp/ (Nishimura et al., 2017). Given that the transcriptome assembly for *P*. cf. *grayi* had the highest TransRate and BUSCO scores (Section 3), all subsequent analyses were conducted using this assembly as the reference.

### 2.5 Phylogenetic analysis

The phylogenetic relationships among the samples were explored using 261 complete and unduplicated single‐copy orthologues (≥180 bp) identified from BUSCO (*N* = 106) and from a reciprocal BLASTX search between the transcriptome assembly of *P*. cf. *grayi* and that of the octocoral *Eunicella verrucosa* (Coelho, M. A. G., Serrão, E. A., Pearson G. A., unpublished data; for additional information about the samples used see Table S1) using DIAMOND (*N* = 155). For the reciprocal blast, we retained contigs with up to five query hits, subsequently selecting only those with a single sequence hit in both results tables (and thus putative single‐copy orthologues). Transcript *.fasta* sequences for orthologues were extracted for each sample from sorted *.bam* files after phasing (samtools phase) and variant calling (bcftools mpileup). Allele sequences were obtained from the resulting *.vcf* files with vcfutils.pl and seqkit fq2fa, aligned with mafft and trimmed with Gblocks in TranslatorX using a custom python script (https://github.com/cymon). The final set of loci used contained no gaps or missing data. The input *.bam* files were generated by mapping reads onto the reference transcriptome using the RSEM (v1.2.31) wrapper script and Bowtie2 (Langmead & Salzberg, 2012; Li & Dewey, 2011). Locus alignments were assigned to “pseudoalleles” (i.e. alleles derived from in silico phasing of short read data stored in the alignment files) and analyzed by maximum likelihood in IQ‐TREE 2 (Minh, Schmidt, et al., 2020). A partitioned analysis was performed by fitting a separate evolutionary model of sequence evolution for each locus using ModelFinder and based on BIC score. Gene and site concordance factor (CF) analysis, which estimates the percentage of gene trees (gCF) or sites (sCF) which agree with the consensus species tree, was performed following Minh, Hahn, and Lanfear (2020) and the recommendations at http://www.iqtree.org/doc/.

Species delimitation on the same multi‐individual dataset as used for the species tree was performed using Species bOundry Delimitation using Astral method (SODA; Rabiee & Mirarab, 2020) based on quartet tree analysis with ASTRAL‐III (v5.7.3; Rabiee et al., 2019; Zhang et al., 2018). The input ML gene trees from IQ‐TREE 2 were used, and a species tree was estimated with ASTRAL‐III. SODA uses the mapping of quartet tree constellations onto an ASTRAL species tree with all individuals to assess if/where coalescence of the tree topology is completely random. Where the coalescence is not random, species boundaries are defined based on the quartet frequencies (Rabiee & Mirarab, 2020).

In addition to SODA species delimitation, we reconstructed the species tree under an ML polymorphism‐aware model (PoMo; De Maio et al., 2015). PoMo is a statistically consistent method for species tree estimation that builds on nucleotide substitution models to incorporate population‐level processes from allele frequency information (Borges & Kosiol, 2020). PoMo was run in IQ‐TREE 2 under the HKY (Hasegawa‐Kishino‐Yano) substitution model, using individual allele count data from the 261 single‐copy orthologue alignments. For the analysis, individual counts were assigned to a total of eight populations corresponding to sampling location and color morph. Branch support values were estimated with 1000 ultrafast bootstrap replicates. The species *Paramuricea biscaya* was used as an outgroup in all the analyses. We downloaded RNA‐seq data for four samples from NCBI's SRA (Accessions: SRX4389727‐SRX4389730; DeLeo et al., 2018). Sequence data were filtered and mapped as described above.

Finally, to extend the taxonomic coverage of the analysis to other *Paramuricea* spp., we conducted an ML tree reconstruction in IQ‐TREE 2 with the mitochondrial gene *mt‐mutS*. Sequence data were retrieved from NCBI Genbank and from *.bam* files generated here by mapping the RNA‐seq reads onto the reference mitogenome of *P. clavata* using RSEM (Genbank Accession: NC_034749) for a subset of the samples (for additional information see Figure S1). New *mt‐mutS* sequences were deposited in NCBI GenBank (Accession numbers: ON804207‐ON804214).

### 2.6 Population differentiation based on microsatellite loci

#### 2.6.1 Sample collection

Sampling in the Atlantic took place between 2010 and 2013 in Portugal. The occurrence of *P*. cf. *grayi* (heretofore *Paramuricea* aff. *clavata*) along the southern coast of Portugal was known from deep divers' records, from shallow hard bottoms down to 90 m depth (Boavida, Assis, et al., 2016; Boavida, Paulo, et al., 2016). We extended the search along the Portuguese west coast between 30 and 70 m depth with the support of volunteer divers (through the citizen participation project Deep Reefs). Nine locations were sampled: Berlengas archipelago (2 sites 20 km apart), Cape Espichel (2 sites 400 m apart), Sines (1 site), Sagres (2 sites 300 m apart), Lagos (1 site), and Tavira (1 site; Table S1 and Figure 1). At each site, samples were collected haphazardly taking well‐separated colonies (i.e., individuals) of the same height (~30 cm height, adult colonies) to avoid sampling the same colony multiple times and to account for the very low rate of asexual reproduction observed in *P. clavata/P*. cf. *grayi* (Coma et al., 1995; Pilczynska, Boavida, et al., 2017). At each site, sample size varied from 12 (BAL) to 84 (CAT) individuals according to the species' density and decompression time constraints in deeper dives (Table S1). Samples consisted of 10 cm apical branches that were preserved in 96% ethanol at room temperature until DNA extraction. Two additional locations in the Mediterranean were sampled (VAC and PZU; Table S1) and combined with 35 existing sampling locations in the Mediterranean Sea described by Mokhtar‐Jamaï et al. (2011). Overall, the dataset covered 46 locations, nine from the Atlantic and 37 from the Mediterranean, and included 1597 individuals (448 newly sampled; Figure 1 and Table S1).

#### 2.6.2 DNA extraction, amplification and genotyping

Genomic DNA of the Atlantic samples was extracted with a cetyltrimethylammonium bromide protocol (CTAB; Winnepenninckx et al., 1993), with purification by standard chloroform:isoamyl alcohol (24:1) followed by DNA precipitation. All samples (*N* = 384 colonies) were genotyped at six microsatellite loci: Parcla_09, Parcla_10, Parcla_12, Parcla_14, Parcla_17 (Molecular Ecology Resources Primer Development Consortium, 2010) and Par_d (Agell et al., 2009). The dataset from Mokhtar‐Jamaï et al. (2011) and the additional Mediterranean sites (*N* = 1243 samples) were extracted using a salting out procedure (Mokhtar‐Jamaï et al., 2011). All samples were genotyped as in Mokhtar‐Jamaï et al. (2011) with minor modifications of the PCR protocol (for more details see Appendix S1). After quality filtering, the microsatellite dataset kept for downstream analyses included 1223 samples from 46 sites (filters: 23% of samples excluded due to missing data, sites with <5 samples and identical multilocus genotypes).

#### 2.6.3 Analysis of genetic diversity among lineages

Indices of genetic diversity were compared among the three lineages identified with SODA, *P. clavata* from the Mediterranean and two segregating lineages of *P*. cf. *grayi* coincident with color morphotype (yellow or purple) within the Atlantic (see Results). We compared the observed heterozygosity (*H*_o_), gene diversity (*H*_e_; Nei, 1973), *F*_IS_ (Weir & Cockerham, 1984), allelic richness (*Ar*_g_) and private allelic richness (*Ap*_g_). The *H*_o_, *H*_e_ and *F*_IS_ were computed for each lineage with GENETIX 4.05 (Belkhir et al., 2004), whereas *Ar*_g_ and *Ap*_g_ were estimated using the rarefaction method (Petit et al., 1998) implemented in ADZE (Szpiech et al., 2008). Statistical support for differences in *Ar*_g_ and *Ap*_g_ were tested with a Kruskal–Wallis rank sum test followed by pairwise Wilcoxon rank sum tests in R. Further details about these procedures can be found in Appendix S1.

#### 2.6.4 Genetic structure

A Bayesian clustering analyses was conducted with STRUCTURE 2.3.4 (Pritchard et al., 2000) to evaluate the number of genetic clusters (*K*) from the individual genotypes without assumptions concerning population boundaries. Because of the unbalanced sampling (Wang, 2017), we considered the admixture model with uncorrelated allele frequencies, a separate ALPHA for each population and an initial ALPHA value = 0.022 (1/46 the number of sampling locations). Null homozygous genotypes were considered as missing data (Falush et al., 2003, 2007). Ten independent runs were performed for each *K* ranging between 1 and 30, with a burn‐in period of 1,000,000 followed by 250,000 iterations and using the StrAuto pipeline for parallelization (Chhatre & Emerson, 2017). The *K* value corresponding to the “upper most hierarchical level of structure” was determined using the likelihood of observing the data (ln*P*(*D*)). CLUMPP 1.1 (Jakobsson & Rosenberg, 2007) and DISTRUCT 1.1 (Rosenberg, 2004) were used for graphical output.

A discriminant analysis of principal components (DAPC; Jombart et al., 2010) was conducted in ADEGENET (Jombart, 2008). Data were transformed into principal components and discriminant analyses were used to maximize variation among‐groups while minimizing within group variation. Population locations were used as a group prior. Based on the *a‐score* method, the number of principal components was set to 80, while we retained two discriminant functions. Hierarchical genetic subdivision (i.e., among lineages, among population and among individuals) was analyzed with an analysis of molecular variance (AMOVA; Excoffier et al., 1992; Michalakis & Excoffier, 1996) based on the number of different alleles (infinite allele model) as implemented in GenAlex 6.51 (Peakall & Smouse, 2012) and using 999 permutations. Total and pairwise *G*_ST_s and *D*_EST_s (Jost, 2008) among lineages and populations were estimated in GenoDive (Meirmans & van Tienderen, 2004). These two measures provide complementary information about genetic structure. Indeed, the *G*_ST_ can be considered as an estimate of the nearness to fixation while *D*_EST_ provides information about the level of allelic differentiation (Jost et al., 2018). Significance was tested with a permutation procedure (*n* = 1000) for the two measures.

The evolutionary relationships among populations were characterized using the chord distance (*D*_c_; Cavalli‐Sforza & Edwards, 1967). Allele frequencies were bootstrapped 100 times in SEQBOOT in PHYLIP 3.6 (Felsenstein, 2005). The consensus tree was obtained using GENDIST, NEIGHBOR and CONSENSUS in PHYLIP and drawn with FigTree v.1.4.4 (https://github.com/rambaut/figtree).

## 3 RESULTS

### 3.1 Phylogenomic inference and species delimitation using RNA‐seq data

#### 3.1.1 Transcriptome sequencing and quality of the assemblies

RNA‐sequencing generated a global dataset of 143 Gb high‐quality paired‐end reads (28.04 ± 0.91 M reads per sample [mean ± SE]), 38 and 105 Gb for *P*. cf. *grayi* and *P. clavata*, respectively (Table S2). Quality assessment of the four de novo transcriptome assemblies obtained for each population showed that those built for *P*. cf. *grayi* (Baleeira, Portugal) and the Italian population of *P. clavata* (Altare) were superior to those obtained for the other two *P. clavata* populations (La Vaca and Balun; Table S3). Transrate quality metrics for the two top assemblies were generally similar (average Transrate contig score = 0.36 for *P*. cf. *grayi* and 0.33 for *P. clavata*; Table S3). BUSCO analysis of metazoan single‐copy orthologues indicated that the curated assemblies (i.e., following ORF clustering and filtering of non‐Anthozoa sequences) from both the Baleeira and Altare populations were very complete, with 98% and 97.4% of core proteins detected, respectively (Table S3). After curation, a total of 73,871 contigs were assembled in *P*. cf. *grayi* (Baleeira population), with an N50 of 1452 bp (Table 1).

**TABLE 1:** Summary statistics for the top transcriptome assembly obtained with rnaSPAdes using samples of *Paramuricea* cf. *grayi* from Baleeira, Portugal (NE Atlantic).

Metric	*P*. cf. *grayi*
Original contigs (rnaSPAdes)	524,051
Good contigs (Transrate)	482,166
ORFs (FragGeneScan)	688,540
Clustered ORFs (VSEARCH)	544,317
Final contigs (Anthozoa top hits)	73,871
Longest sequence (nt)	48,483
Shortest sequence (nt)	108
Mean sequence length (bp)	969
Median sequence length (bp)	654
N50 (bp)	1452
Sequences >1k bp	24,579 [33.3%]
Sequences >10k bp	44 [0.1%]
G‐C content (%)	45.4

#### 3.1.2 Phylogenetic analysis

**FIGURE 2:**
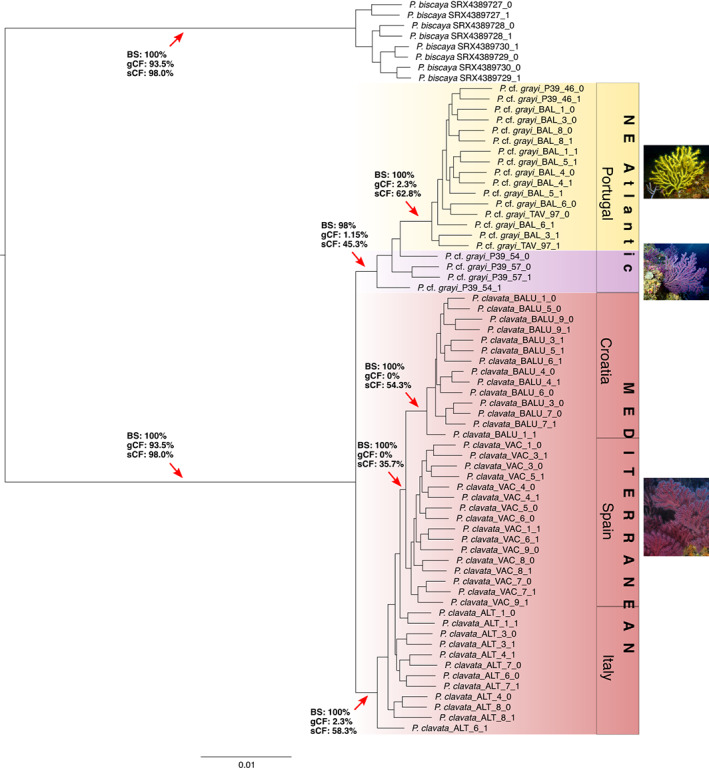
Phylogenetic relationships in *Paramuricea* determined by ML partition analysis in IQ‐TREE 2. The tree was built using an alignment of 261 single‐copy orthologues with a separate evolutionary model per gene (see text for details). The colors correspond to species identified by SODA analysis (plus outgroup, *P. biscaya*); text boxes to the right indicate geographic region from which samples originate. Red arrows indicate selected key branches with ultrafast bootstrap support (1000 replicates); gCF/sCF = gene/site concordance factors supporting the branch indicated. For the NE Atlantic, the species name is given as *P*. cf. *grayi* with color morphotypes indicated in yellow and purple. The codes of the sites from which the samples originate are embedded in the taxon names (P39, BAL, TAV, VAC, ALT and BALU) and follow the nomenclature used in Figure 1 and Table S1. Please note that there are two terminal nodes for each specimen, one for each of two multi‐locus "pseudoalleles" (i.e. alleles derived from in silico phasing of the short read data). The pseudoalleles are distinguished by either a zero or a one appended to the taxon name. Photo credits: A. C. Ferreira (*P*. cf. *grayi*) and J. Garrabou (*P. clavata*).

Our partitioned ML analysis of 261 single‐copy orthologues clearly separated the Atlantic (southern‐central Portugal) *P*. cf. *grayi* and the Mediterranean *P. clavata* samples with high (Atlantic clade, 98%) or full (Mediterranean) support (Figure 2). Within the Atlantic samples, the yellow morphotype of *P*. cf. *grayi* from three distinct locations (including P39 off Cape Espichel where both color morphs occur in sympatry) formed a monophyletic group to the exclusion of the purple color morph (Figures 1 and 2). Within the Mediterranean, individuals from Croatia (Adriatic Sea) also formed a fully‐supported clade with full support and distinct from the Spanish and Italian samples (Figure 2).

Despite the high bootstrap support for the Atlantic and Mediterranean clades, concordance analysis of the branches supporting these clades revealed very low gCFs of only 1.15% (Atlantic) and 2.3% (Mediterranean). Concordance factors for sites (sCF) were also low to moderate (45.3% and 58.3%), which together with the short branch lengths supporting these clades, suggests recent divergence with incomplete lineage sorting (ILS). We note, however, that the branch supporting the yellow morphotype clade within the Atlantic samples was somewhat longer than those supporting the Atlantic and Mediterranean clades, had full bootstrap support, and a fairly robust sCF (62.8%; Figure 2).

#### 3.1.3 Species delimitation

**FIGURE 3:**
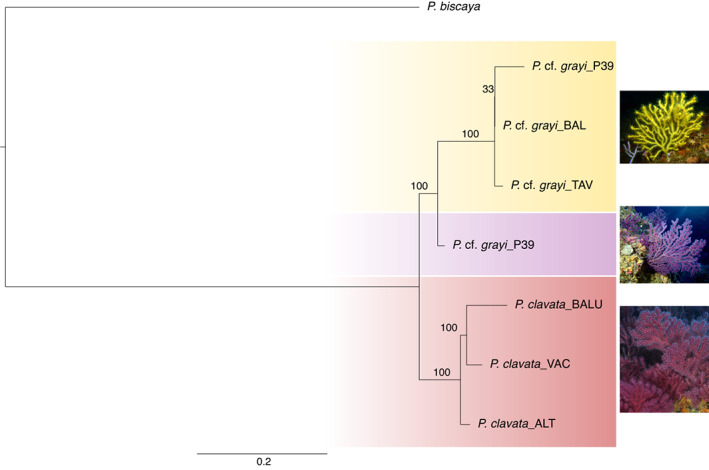
Species tree of *Paramuricea* samples in this study using the ML polymorphism‐aware (PoMo) model and HKY substitution model in IQ‐TREE 2. The tree was built using allelic counts from 261 single‐copy orthologues. Values on the branches indicate ultrafast bootstrap support (1000 replicates). The colors correspond to species identified by SODA analysis (plus outgroup, *P. biscaya*). For the NE Atlantic, the species name is given as *P*. cf. *grayi* with color morphotypes (lineages) indicated in yellow and purple. The codes of the sites from which the samples originate are embedded in the taxon names (P39, BAL, TAV, VAC, ALT and BALU) and follow the nomenclature used in Figure 1 and Table S1. Photo credits: A. C. Ferreira (*P*. cf. *grayi*) and J. Garrabou (*P. clavata*).

Species delimitation based on the ASTRAL‐III species tree and performed with the SODA software supported the clades formed by samples of *P*. cf. *grayi* and *P. clavata* as separate species (Figure 2). The same split was also evident with the barcode marker *mt‐mutS* for which our analyses recovered two distinct haplotypes, one including all *P. clavata* samples from the Mediterranean (96% bootstrap support) and another including the samples of *P*. cf. *grayi* sequenced here together with *P. grayi* from the northern Iberian Peninsula (100% bootstrap support; Figure S1). Furthermore, within the Atlantic, the purple and yellow morphotypes of *P*. cf. *grayi* were all recovered as two distinct species in the SODA analysis (with the caveat that our dataset only included two purple individuals, although they were sympatric with one individual of the yellow morph – site P39). These results were congruent with the PoMo ML species tree, which supported the Mediterranean (*P. clavata*) and Atlantic (yellow and purple morphotypes of *P*. cf. *grayi*) populations as three distinct entities with full bootstrap support (Figure 3). Given that our sampling included just a few individuals of the purple morphotype, we conservatively assign both color morphs of the Atlantic clade to *P*. cf. *grayi*, a species first described (as *Acanthogorgia grayi*) from Madeira, Portugal (Johnson, 1861), but which has also been documented to occur in southern Portugal (Grasshoff, 1977, 1992; Monteiro‐Marques, 1987). This species identification is thus based on molecular data presented here and elsewhere (Poliseno et al., 2017), as well as on historical taxonomic records documenting *P. grayi* (but not other *Paramuricea* species) at our sampling sites in Portugal.

### 3.2 Population genetics based on microsatellite loci

#### 3.2.1 Genetic variability

The observed (*H*_o_) and expected (*H*_e_) heterozygosity ranged between 0.44 (TAV) and 0.83 (PZO; mean ± SD = 0.66 ± 0.08) and between 0.5 (BAL) and 0.8 (PHL; mean ± SD = 0.71 ± 0.07), respectively. Multilocus *F*_IS_ were between 0 (e.g., VAC, LAG, BAL) and 0.3 (TAV; mean ± SD = 0.07 ± 0.08). Significant heterozygote deficiencies, after FDR correction, were found in 15 of 46 populations (28%), located both in the Atlantic (e.g., TAV, SIN) and in the Mediterranean (e.g., CAS, RIO). Regarding rarefied allelic (*Ar*_(18)_) and private allelic richness (*Ap*_(18)_), we found highly contrasting values among populations. *Ar*_(18)_ ranged between 3.5 (BAL) and 7.92 (PHL; mean ± SD = 6.51 ± 1.1), while *Ap*_(18)_ was null for 14 populations (all belonging to the Mediterranean, e.g., CPS, CLB, MES) with a maximum of 1.52 for TAV (mean ± SD = 0.17 ± 0.3). Seven of the highest *Ap*_(18)_ values were observed for Atlantic populations (LAG, BAL, SEG, SIN, RAB; Table S4). Significant differences among the three lineages (see below) were observed for all the genetic diversity parameters (*H*_o_, *H*_e_, *Ap*_(18)_, *Ar*_(18)_) with the exception of *F*_IS_. Pairwise Wilcoxon rank tests showed that significant differences were mostly driven by variation between Mediterranean and the Atlantic populations, which included the two segregating lineages identified (see Tables S4 and S5 for details). The highest *H*_o_, *H*_e_ and *A*_r_ values were observed in *P. clavata*, whereas the yellow lineage of *P*. cf. *grayi* harbors the highest *A*_p_.

#### 3.2.2 Population structure

**FIGURE 4:**
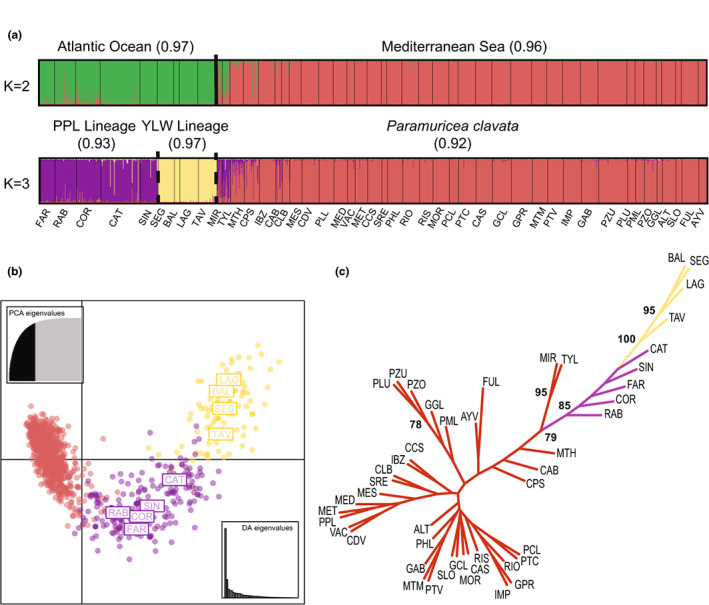
Spatial genetic structure in *Paramuricea*: (a) clustering analysis conducted with STRUCTURE considering *K* = 2 and 3. Each individual is represented by a vertical line partitioned in *K*‐colored segments, which represent the individual membership fraction in *K* clusters. Thin black vertical lines delineate the different locations of the samples while dashed and thick lines show the segregating lineages of *P*. cf. *grayi* (PPL for ‘purple’ and YLW for ‘yellow’; see results below) and the two oceanographic basins (Atlantic vs. Mediterranean), respectively. Samples names are shown below the assignment plots (for abbreviations see Table S1). The mean membership coefficient for each cluster is also shown. (b) Scatter plot of the discriminant analysis of principal components (DAPC) based on an a‐score of 80 and considering axes 1 and 2. These two axes represented 51.1% of the total variation in the data. Each dot corresponds to one individual (*n* = 1223) from each of the 46 locations. Note that the colors correspond to the three clusters with the red dots for the Mediterranean samples of *P. clavata* and the yellow and purple dots for the Atlantic samples of *P*. cf. *grayi*. Only the names of the Atlantic samples are shown. (c) Dendrogram based on the chord distance (Cavalli‐Sforza & Edwards, 1967) showing the relationships among the 46 populations. Colors are the same as for *K* = 3 while the sample names are shown in Table S1. Only bootstrap values higher than 75 are shown.

The pattern of population structuring was largely congruent with the phylogenetic analyses. The ln*P*(*D*) parameter increased until *K* = 4, plateaued until *K* = 9 and decreased for higher *K* values (Figure S2). Considering our focus on the genetic structure among Atlantic and Mediterranean populations, we describe only the results for *K* = 2 and 3. At *K* = 4, structuring among Mediterranean populations became apparent, which has been previously characterized (Mokhtar‐Jamaï et al., 2011). For *K* = 2, individuals from the Atlantic (purple and yellow lineages; mean membership coefficient = 0.97) were separated from those from the Mediterranean (mean membership coefficient = 0.96; Figure 4a), with a signal of admixture between the purple lineage of *P*. cf. *grayi* and the Mediterranean populations of *P. clavata* close to the Strait of Gibraltar (MIR, TYL and MTH), and extending to southern Spain (CPS) and the Balearic Islands (CAB). The three genetic clusters (*K* = 3) were concordant with the areas corresponding to western (*P*. cf. *grayi* purple lineage; mean membership coefficient = 0.93) and southern (*P*. cf. *grayi* yellow lineage; mean membership coefficient = 0.97) Portugal and the Mediterranean (*P. clavata*; mean membership coefficient = 0.92; Figure 4a).

The DAPC analysis corroborates the results obtained with STRUCTURE. The first two axes represented relatively high levels of the total variation, 40.4% and 10.7%, respectively. The first axis separated *P. clavata* populations from the Mediterranean and *P*. cf. *grayi* populations from the Atlantic (purple and yellow lineages), while the second axis divided the two lineages of *P*. cf. *grayi* (Figure 4b). The topology of the tree based on the Chord distance (*D*_c_) supported the distinction between the Atlantic and the Mediterranean populations with a bootstrap value of 85 (Figure 4c).

The AMOVA revealed highly significant genetic structuring between the three groups (i.e. lineages), among populations, and among individuals (*p* < .001; Table S5). The genetic variation explained by differences among groups was 14%, higher than that explained by differences among populations (10%) or among individuals (6%). The global *G*_ST_ and *D*_EST_ were 0.17 and 0.53, respectively (*p* < .001). Pairwise *G*_ST_ among lineages were 0.24, 0.12, and 0.11 for *P. clavata* vs. yellow lineage of *P.* cf. *grayi*, yellow vs. purple *P*. cf. *grayi* lineages and *P. clavata* vs. purple lineage of *P.* cf. *grayi*, respectively. Overall, pairwise *G*_ST_ ranged between 0.01 (PPL vs. MED) and 0.46 (AYV and BAL), with only 31 nonsignificant comparisons (<3% of total; Table S6). Pairwise *D*_EST_ ranged between 0.01 (PPL vs. MED) and 0.99 (AYV and SEG). All except 33 pairwise comparisons (<5%) were significant. Focusing on the Atlantic populations, the values of the pairwise *G*_ST_ and *D*_EST_ were within the range of the values observed among Mediterranean populations (0.04 [SEG vs. BAL] to 0.31 [FAR vs. BAL] for *G*_ST_; 0.06 [SEG vs. BAL] to 0.67 [RAB vs. BAL] for *D*_EST_). For the two estimators, all but one pairwise comparison (SEG vs. BAL) were significant.

## 4 DISCUSSION

This study discovered novel species diversity in the gorgonian genus *Paramuricea* in the Iberian Peninsula, revealing recently diverged species found predominantly in the Mediterranean (*P. clavata*) and in the Atlantic (*P*. cf. *grayi*), where two color morphotypes (yellow and purple) remain genetically distinct in sympatry, indicating also speciation within *P*. cf. *grayi*. These discoveries result from integrating genetic data from three independent types of markers (microsatellites, single‐copy orthologues, and a mitochondrial gene) to assess the extent of divergence between Mediterranean populations of the gorgonian *P. clavata* and adjacent Atlantic populations occurring along southern and western Portugal, suggested to represent the western limit of the species' distribution range (Boavida, Assis, et al., 2016; Cúrdia et al., 2012; Pilczynska et al., 2019). The three phylotranscriptomic approaches used here (ML phylogenetic inference, SODA species delimitation and PoMo species tree reconstruction) strongly support the Atlantic and Mediterranean clades being two distinct sister species: *P*. cf. *grayi* in the Atlantic (shared *mt‐mutS* haplotype with published *P. grayi*), a species previously described for this region (Grasshoff, 1977, 1992; Poliseno et al., 2017); and *P. clavata* in the Mediterranean. Our phylogenetic results are also congruent with genetic structure at microsatellite loci (Pilczynska, Cocito, et al., 2017; this study), with a recent phylogenetic reconstruction based on mitochondrial genomes assembled from RNA‐seq data (Coelho et al., 2022), as well as with a phylogenomic study for *P. grayi*/*P. clavata* collected within the Mediterranean (Quattrini et al., 2022). Although based on a limited number of samples, two statistically consistent species tree methods (SODA, based on the ASTRAL species tree, and PoMo, incorporating within‐population genetic diversity) revealed the genetic segregation of color morphotypes within Atlantic *P*. cf. *grayi*, and thus an additional putative (or ongoing) speciation event between purple and yellow lineages. Although these lineages display considerable geographic structure, with the yellow morphotype largely dominant in the Algarve (south Portugal) and the purple dominating habitats on the west coast of Portugal, they can also be found in sympatry (e.g., site P39).

### 4.1 Lineage divergence across the Atlantic‐Mediterranean transition

Previous genetic studies of Atlantic *P*. cf. *grayi* (heretofore *P. clavata*) occurring along Portugal and Mediterranean populations of *P. clavata* identified high levels of population differentiation between the two regions, indicating highly restricted gene flow across the Atlantic‐Mediterranean transition (Pilczynska et al., 2019; Pilczynska, Cocito, et al., 2017). Based on comprehensive sampling, our microsatellite analyses corroborate these early findings, revealing a clear segregation between *P*. cf. *grayi* and *P. clavata*. The uniqueness of the Atlantic populations of *P*. cf. *grayi* and the relationship with *P. clavata* was also evident in the STRUCTURE and DAPC analyses (Figure 4), which showed a two‐level hierarchical partitioning of genetic variation that agreed with the results of the phylogenetic analyses based on single‐copy ortholog alignments (Figures 2 and 3), as well as with the statistically significant differences at most genetic parameters analyzed. Private allelic richness (*Ap*_(18)_), in particular, was substantially higher in *P*. cf. *grayi*, which included seven of the highest *Ap*_(18)_ values observed across all populations studied. This pattern is more consistent with the long‐term persistence of distinct lineages, rather than Atlantic populations being derived as marginal extensions of a Mediterranean expansion. Indeed, all phylogenetic methods, as well as a distance‐based approach, indicate that Mediterranean *P. clavata* is more genetically divergent from the adjacent Atlantic populations of *P*. cf. *grayi* (yellow lineage) than from the more geographically distant populations in western Portugal (purple lineage).

Although our analyses did not consider morphological data, the ML analysis of the *mt‐mutS* barcode showed that the samples of the Atlantic lineage(s) identified here have the same haplotype as the morphospecies *P. grayi* from northern Iberia (Galicia and Bay of Biscay; Poliseno et al., 2017) and distinct from that of the sister species *P. clavata* (Figure S1). Thus, while we cannot formally reject the occurrence of *P. clavata* in adjacent Atlantic coral assemblages (e.g., unsampled populations in northern Morocco), our results confirm the identity of all southern and western Iberian populations studied here as *P*. cf. *grayi*. This finding agrees with previous morphological identifications documenting the presence of *P. grayi* in southern Portugal, particularly in circalittoral rocky habitats along the Algarve and in the Gorringe Bank (Grasshoff, 1977, 1992; Monteiro‐Marques, 1987).

The sister relationship between *P. grayi* and the Mediterranean endemic *P. clavata* has been recently described in two studies. Based on *mt‐mutS* sequence data, Poliseno et al. (2017), estimated that the divergence between the two species occurred 2.6 Ma (4–1 Ma) or 4.6 Ma (7–3 Ma) assuming a mutation rate of 0.14% or 0.25% Myr‐1, respectively. The authors suggest that speciation occurred through vicariance in association with the Messinian (5.96–5.33 Ma) and/or Gelasian (2.6 Ma) crises (Krijgsman et al., 1999; Por, 2009). Indeed, the diversification of marine taxa in the Mediterranean is thought to be greatly influenced by these two events, and by the subsequent climatic oscillations during the Quaternary, contributing to the high biodiversity and endemism of present‐day Mediterranean fauna and flora (Bianchi et al., 2012; Bianchi & Morri, 2000; Lejeusne et al., 2010).

More recently, Quattrini et al. (2022) used RAD‐seq data to estimate the timing of divergence between *P. clavata* and *P. grayi* at 2 Ma (3.3–1.0 Ma 95% CI), with Bayesian dispersal‐extirpation‐cladogenesis modeling suggesting that divergence likely occurred in sympatry (though the analysis only included three individuals collected within the Mediterranean). Our STRUCTURE analysis based on microsatellite loci identified a signal of admixture between *P*. cf. *grayi* and *P. clavata* at sites in the Alboran sea (MIR, TYL, MTH, located immediately east of the Strait of Gibraltar). Here and in two additional sites east of the AOF (CPS and CAB), the coefficients of membership to either of the Atlantic/Mediterranean clusters were generally lower (Figure 4a). This suggests that the Alboran sea is an area where the two species come into secondary contact, presumably following vicariant speciation. Interestingly, the admixture signal indicates that introgression occurs predominantly from the Atlantic (i.e., *P*. cf. *grayi*) into the Mediterranean (i.e., *P. clavata*) gene pool, and involves alleles found in the purple lineage of *P*. cf. *grayi* (Figure 4a). However, this inferred directionality could be a sampling artifact, since Atlantic samples from the adjacent northern Moroccan coastline are currently unavailable. Such a pattern of admixture has been described in other Atlantic‐Mediterranean sister taxa/lineages (Bermejo et al., 2018; Duranton et al., 2019, 2020; Tine et al., 2014; but see Alberto et al., 2008), and can be explained by the inflow of Atlantic surface seawater into the Mediterranean resulting from water balance deficits inherent during interglacial periods, which contributes to the continued penetration of Atlantic fauna and flora (Bianchi et al., 2012; Harmelin & D'Hondt, 1993). While further research is needed to fully resolve the evolutionary history of *P. grayi* and *P. clavata* in the Atlantic‐Mediterranean, our findings seem to support suggestions that *P. clavata* is a “neoendemic” species that diverged from a subtropical Atlantic ancestor that colonized the Mediterranean following the Zanclean flood (Bianchi et al., 2012). The divergence ages estimated recently (Poliseno et al., 2017; Quattrini et al., 2022) suggest that the Alboran sea (through the Strait of Gibraltar and AOF) has acted as a buffer reducing admixture between *P*. cf. *grayi* and *P. clavata* throughout the Quaternary, as documented previously for other taxa as a consequence of the coupling between oceanographic barriers (here AOF) and genetic barriers (Ayari et al., 2019).

The low and moderate values of concordance factors obtained for genes (gCFs) and sites (sCFs) supporting the Atlantic and Mediterranean clades provide further evidence for a recent divergence between *P. clavata* and *P*. cf. *grayi* (Figure 2). Gene‐tree discordance is generally attributed to two processes: (1) incomplete lineage sorting (ILS) of ancestral polymorphisms; and (2) gene flow between taxa via introgressive hybridization (Maddison, 1997; Maddison & Knowles, 2006; Suh et al., 2015; Yu et al., 2013). Introgression is considered a pivotal process countering lineage diversification of both plants and animals, which is often observed in rapidly (or recently) diverging lineages of closely related species (Berner & Salzburger, 2015; Mallet, 2007; Seehausen, 2004), including several coral lineages (Combosch & Vollmer, 2015; Mao et al., 2018; Poliseno et al., 2021; Quattrini et al., 2019; van Oppen et al., 2001; Vollmer & Palumbi, 2002). Disentangling ILS and introgressive hybridization is often difficult because they generate similar genomic signatures (Meyer et al., 2017). However, the congruency between species tree methods (i.e., concatenation and statistically consistent ASTRAL and PoMo models) and between phylogenies based on nuclear and mitochondrial loci analyzed here, as well as evident admixture in the Alboran contact zone suggest that ILS is a more likely explanation for the overall low values of gCFs and sCFs supporting the *P*. cf. *grayi* and *P. clavata* clades (Toews & Brelsford, 2012; Twyford & Ennos, 2012).

### 4.2 Genetic segregation within the Atlantic lineages

Our results clearly show lineage divergence within Iberian *P*. cf. *grayi*. The microsatellite analysis revealed that populations in southern Portugal (Sagres, Lagos and Tavira) were highly differentiated from those sampled along the western coast (Berlengas, Cape Espichel and Sines), with highly significant pairwise *G*_ST_s and *D*_EST_s and genetic partitioning (Table S6 and Figure 4). This south‐west genetic divide is coincident with the distribution of yellow and purple color morphotypes noted in previous studies examining a subset of the populations (Pilczynska et al., 2019; Pilczynska, Cocito, et al., 2017). Importantly, our ML and species trees support color morph segregation in *P*. cf. *grayi* irrespective of geographic origin, including sympatric individuals (P39 site, Figures 1, 2, 3), which is important preliminary evidence for a diagnostic morphological trait in distinguishing the two Atlantic lineages. It is noteworthy that our STRUCTURE analysis identified a signal of admixture between the two lineages at sites where field surveys confirmed them to occur in sympatry (CAT and SIN; Figure 4a). Collectively, these findings and observations suggest a high degree of habitat segregation (and partial reproductive isolation) between the two morphotypes of *P*. cf. *grayi*, calling for additional analyses using genomic data, broader taxon and habitat (e.g., depth) sampling, as well as experimental approaches (e.g., crossing experiments) to clarify the taxonomic status of the two color morphs.

Within segregating lineages of *P*. cf. *grayi*, we have identified significant pairwise genetic differentiation between most populations studied, including at local spatial scales, from ~20 km (e.g., BAL vs. LAG, southern Portugal) to less than 400 m (e.g., COR vs. CAT, western Portugal; Table S6). These results suggest restricted gene flow between populations of both Atlantic lineages, with rare long‐distance dispersal. The extent to which such genetic differentiation is driven by seascape (i.e., physical environment), life‐history traits of the species or the interplay between both remains speculative at this point. However, these findings raise several important questions about the biology of *P*. cf. *grayi*. For instance, strong genetic differentiation at small spatial scales and a pattern of isolation by distance are common in many brooding corals in which dispersal potential is generally low given the short duration of the pelagic larval stage and predominantly philopatric recruitment (e.g., Ayre & Hughes, 2000; Gazulla et al., 2021; Ledoux, Garrabou, et al., 2010; Mokhtar‐Jamaï et al., 2011; Smilansky & Lasker, 2014; Underwood et al., 2007; but see discussions in Coelho & Lasker, 2016b; Jones et al., 2009). Interestingly, the spatial genetic patterns described for *P. clavata* in the Mediterranean (Arizmendi‐Mejía et al., 2015; Ledoux et al., 2018; Mokhtar‐Jamaï et al., 2011, 2013) coincide with the low effective dispersal inherent to surface brooding inferred from field observations and parental genetic analysis (Coma et al., 1995; Mokhtar‐Jamaï et al., 2013; but see Guizien et al., 2020), while future studies could indicate whether *P*. cf. *grayi* is also a brooder. We note, however, that reproductive mode in octocorals is relatively plastic even at the congeneric level (e.g., *Antillogorgia*: Coelho & Lasker, 2014; Coelho & Lasker, 2016a; Gutiérrez‐Rodríguez & Lasker, 2004; and *Alcyonium*: McFadden et al., 2001; reviewed in Kahng et al., 2011).

## 5 CONCLUDING REMARKS

In this study, we investigated population differentiation and speciation in key structural species of octocorals in marine ecosystems of the NE Atlantic and Mediterranean Sea. The results presented advance the understanding of lineage diversification in these important biogeographic regions. Our analyses revealed that past species identifications based on morphological characters and a limited number of genetic markers have underestimated species diversity in *Paramuricea* occurring in shallow‐water and circalittoral habitats of southern Iberia. This has important implications for conservation planning, as misidentifying putative species as isolated populations compromises the effectiveness of the adopted measures. Specifically, our results provide compelling evidence defining species boundaries between Atlantic populations, here assigned to the morphospecies *P*. cf. *grayi*, and the Mediterranean *P. clavata*; as well as between two segregating lineages within the *P*. cf. *grayi* clade that are coincident with coloration of the colonies, thus casting doubts about the taxonomic status of these two entities. While the conservation status of *P. grayi* remains unassessed, *P. clavata* has been listed as vulnerable in the IUCN red list of anthozoans in the Mediterranean since 2016 (Otero et al., 2017).

Although the broader species limits between *P*. cf. *grayi* and *P. clavata* could be resolved with a single mitochondrial barcode (*mt‐mutS*), this study emphasizes the need for genome‐wide multi‐locus datasets to resolve recent lineage splitting events in the face of, for example, ILS and/or introgression. The marked spatial genetic structure and differentiation observed between the *P*. cf. *grayi*/*P. clavata* clades reveal historical footprints of divergence across the Atlantic‐Mediterranean transition, the corresponding evolutionary scenarios of which could be tested with further population genomic data. Like recent studies, our results emphasize the urgent need to re‐evaluate species diversity and the processes underlying diversification within the genus *Paramuricea* (Coelho et al., 2022; Poliseno et al., 2017; Quattrini et al., 2022; Thoma, 2013), which despite its ecological importance, likely harbors high levels of cryptic diversity (e.g., several species described as amphi‐Atlantic; Sampaio et al., 2019; Walting et al., 2011). Finally, cryptic diversity that went undetected in previous molecular and morphological studies validates the usefulness of phylotranscriptomics for resolving the evolutionary relationships of octocorals, and more generally of diverse lineages of marine taxa, including at shallow phylogenetic scales as examined here. Although phylotranscriptomics can be a reliable (and in some cases cheaper) alternative to several emerging approaches based on genome sequence data (e.g., Herrera & Shank, 2016; Quattrini et al., 2017; see discussion in Cheon et al., 2020), it remains largely underutilized in anthozoans (reviewed in Quek & Huang, 2022).

## AUTHOR CONTRIBUTIONS

**Márcio A. G. Coelho:** Conceptualization (equal); data curation (equal); formal analysis (equal); investigation (equal); methodology (equal); resources (equal); validation (equal); visualization (equal); writing – original draft (lead); writing – review and editing (equal). **Gareth A. Pearson:** Conceptualization (equal); data curation (equal); formal analysis (equal); investigation (equal); methodology (equal); validation (equal); visualization (equal); writing – original draft (equal); writing – review and editing (equal). **Joana R. H. Boavida:** Conceptualization (equal); data curation (supporting); formal analysis (supporting); funding acquisition (equal); investigation (equal); methodology (supporting); resources (equal); validation (supporting); writing – review and editing (equal). **Diogo Paulo:** Resources (supporting); writing – review and editing (supporting). **Didier Aurelle:** Formal analysis (supporting); resources (supporting); writing – review and editing (equal). **Sophie Arnaud‐Haond:** Writing – review and editing (equal). **Daniel Gómez‐Gras:** Resources (supporting); writing – review and editing (equal). **Nathaniel Bensoussan:** Resources (supporting); writing – review and editing (supporting). **Paula López‐Sendino:** Investigation (equal); resources (supporting); writing – review and editing (equal). **Carlo Cerrano:** Resources (supporting); writing – review and editing (equal). **Silvija Kipson:** Resources (supporting); writing – review and editing (equal). **Tatjana Bakran‐Petricioli:** Resources (supporting); writing – review and editing (equal). **Eliana Ferretti:** Resources (supporting); writing – review and editing (supporting). **Cristina Linares:** Resources (supporting); writing – review and editing (equal). **Joaquim Garrabou:** Funding acquisition (equal); resources (equal); writing – review and editing (equal). **Ester A. Serrao:** Conceptualization (equal); funding acquisition (equal); writing – review and editing (equal). **Jean‐Baptiste Ledoux:** Conceptualization (equal); data curation (equal); formal analysis (equal); investigation (equal); methodology (equal); resources (equal); validation (equal); visualization (equal); writing – original draft (equal); writing – review and editing (equal).

## Supporting information


Figure S1‐S2.

Appendix S1.

Table S1.
Table S2.
Table S3.
Table S4.
Table S5.
Table S6.

## Data Availability

Raw RNA‐seq data and *mt‐mutS* sequences have been uploaded to NCBI's SRA (BioProject ID: PRJNA847883) and GenBank databases (Accessions numbers: ON804207‐ON804214), respectively. Final sequence alignments and microsatellite data are available on DRYAD (https://doi.org/10.5061/dryad.r7sqv9sfm).
