# Supplementary material for: Not out of the Mediterranean: Atlantic populations of the gorgonian Paramuricea clavata are a separate sister species under further lineage diversification
Source: Ecol Evol. 2023 Jan 29;13(1):e9740. doi: 10.1002/ece3.9740 (PMC9912747; doi:10.1002/ece3.9740)
Supplement: Supplementary file 1 — Figure S1‐S2. [file ECE3-13-e9740-s002.docx]

**Supplemental Information for:**

**Not out of the Mediterranean: Atlantic populations of the gorgonian *Paramuricea* *clavata* are a separate sister species under further diversification**

Márcio A. G. Coelho^1,2*§^, Gareth A. Pearson^1*§^, Joana R. H. Boavida^3^, Diogo Paulo^1^, Didier Aurelle^3,4^, Sophie Arnaud-Haond^5^, Daniel Gómez-Gras^6,7,8^, Nathaniel Bensoussan^3,9^, Paula López-Sendino^9^, Carlo Cerrano^10,11,12,13^, Silvija Kipson^14,15^, Tatjana Bakran-Petricioli^14^, Eliana Ferretti^16,17^, Cristina Linares^7,8^, Joaquim Garrabou^3,9^, Ester A. Serrão^1,18^, Jean-Baptiste Ledoux^19*^

**Table of contents**

Figure S1 – Phylogenetic reconstruction based on *mtMutS* 2

Figure S2 – *K* value plot of the STRUCTURE analysis 3

#
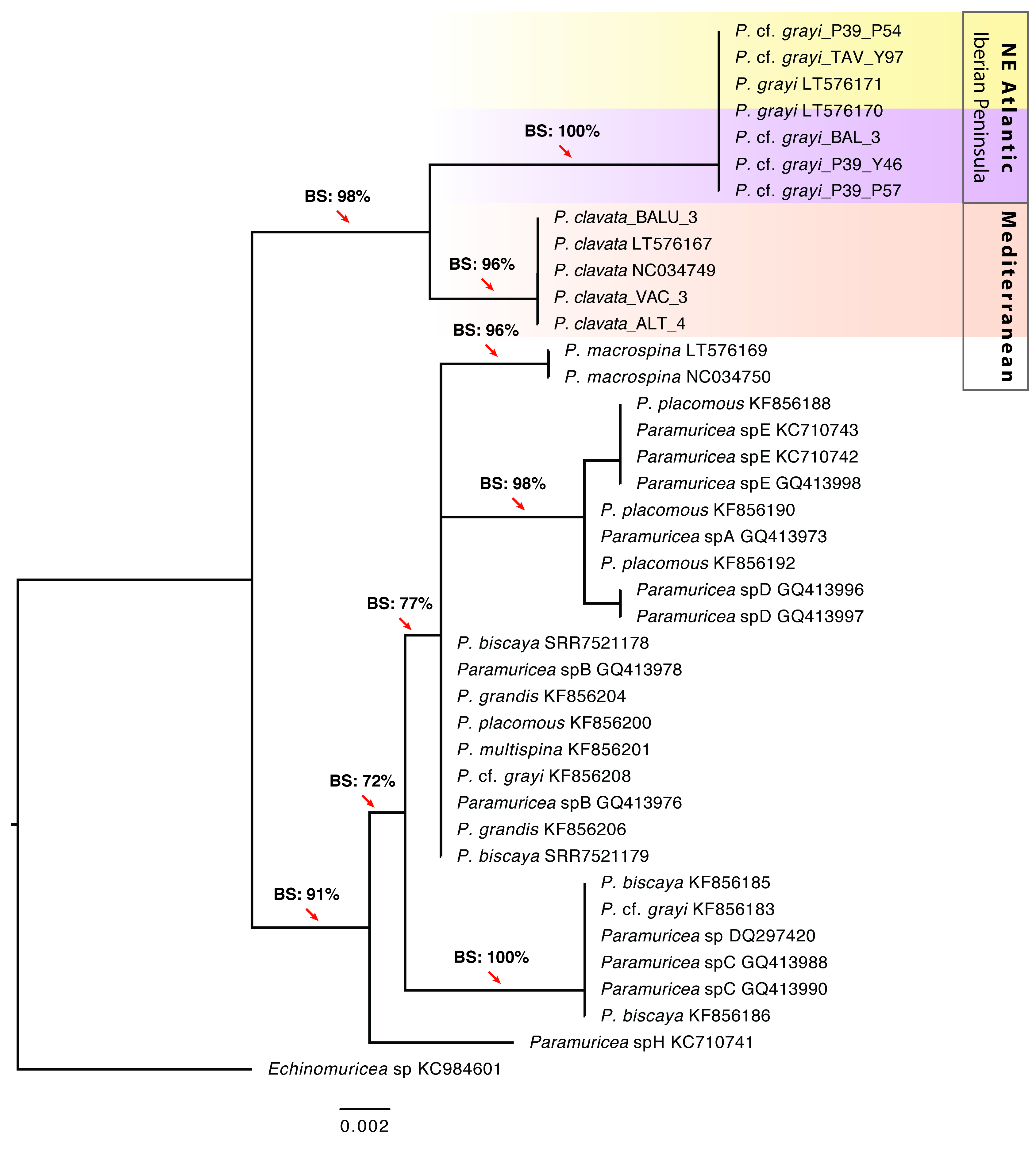
Figure S1 – Phylogenetic reconstruction based on *mtMutS*

Figure S1 - Phylogenetic relationships in Paramuricea determined by ML analysis with IQ-TREE 2. The tree was built using a 684 bp alignment for the mitochondrial gene mt-mutS with the HKY+F substitution model. For the subset of the samples sequenced by RNA-seq, the mt-mutS sequence was obtained by mapping the reads onto the reference mitogenome of P. clavata (Genbank Accession: NC_034749) using RSEM. All downstream analyses, from the generation of a consensus mt-mutS sequence to ML tree reconstruction, were performed as described in the Material and Methods for the transcriptomes (except phasing and variant calling, which were not performed). The assembled sequences were annotated in Geneious Prime v2020.2.4 by importing the annotations of the reference used for read mapping. In total, we assembled the sequence of the mt-mutS gene for 4 samples from each population of Paramuricea studied by Gómez-Gras et al. (in review), the four additional samples collected in Portugal and all the P. biscaya samples used as outgroup in the RNA-seq analyses. Because the mt-mutS sequences were invariant within populations, we did not include all samples in the final mt-mutS ML tree analysis. The colours correspond to species identified by SODA analysis (plus other Paramuricea spp. and the outgroup Echinomuricea sp.). The text boxes to the right indicate geographic region from which samples originate. Please note that the NE Atlantic and Mediterranean clades include two specimens each sequenced elsewhere. Additionally, for the NE Atlantic clade the analysis based on the mt-mutS gene did not resolve the two P. cf. grayi segregating lineages (yellow or purple morphotypes); thus the colour divide does not reflect genetic segregation. Red arrows indicate selected key branches with ultrafast bootstrap support (100 replicates). Accession numbers of sequences retrieved from NCBI GenBank or Sequence Read Archive, as well as the origin of the samples sequenced here (sites P39, BAL, TAV, VAC, ALT and BALU; see Figure 1 and Table S1) are embedded in the taxon names. New mt-mutS sequences were deposited on NCBI's GenBank (Accession numbers: ON804207- ON804214).

# Figure S2 – *K* value plot of the STRUCTURE analysis

Figure S2 - Mean (+/- SD) logarithm of the likelihood of observing the data (lnP(D)) computed over 10 independent runs of STRUCTURE as a function of the number of genetic clusters (K).
