# Supplementary material for: Not out of the Mediterranean: Atlantic populations of the gorgonian Paramuricea clavata are a separate sister species under further lineage diversification
Source: Ecol Evol. 2023 Jan 29;13(1):e9740. doi: 10.1002/ece3.9740 (PMC9912747; doi:10.1002/ece3.9740)
Supplement: Supplementary file 2 — Appendix S1. [file ECE3-13-e9740-s003.docx]

**Supplemental Information for:**

**Not out of the Mediterranean: Atlantic populations of the gorgonian *Paramuricea* *clavata* are a separate sister species under further diversification**

Márcio A. G. Coelho^1,2*§^, Gareth A. Pearson^1*§^, Joana R. H. Boavida^3^, Diogo Paulo^1^, Didier Aurelle^3,4^, Sophie Arnaud-Haond^5^, Daniel Gómez-Gras^6,7,8^, Nathaniel Bensoussan^3,9^, Paula López-Sendino^9^, Carlo Cerrano^10,11,12,13^, Silvija Kipson^14,15^, Tatjana Bakran-Petricioli^14^, Eliana Ferretti^16,17^, Cristina Linares^7,8^, Joaquim Garrabou^3,9^, Ester A. Serrão^1,18^, Jean-Baptiste Ledoux^19*^

**Table of contents**

Note S1 - Supplementary methods 1

Note S1.1. DNA amplification and genotyping 1

Note S1.2. Quality check 1

Note S1.3. Population and lineage genetic diversity 1

Note S2 – Supplementary results 2

Note S2.1. Genetic variability at microsatellite loci 2

Note S3 - Supplementary references 2

# Note S1 - Supplementary methods

## Note S1.1. DNA amplification and genotyping

All samples were genotyped in the same conditions as in Mokhtar-Jamaï et al. (2011) with minor modifications for the PCR protocol (1.5 mM MgCl2, cycle: 95ºC 3 min, 94ºC 20 sec, 45ºC 20 sec, 72ºC 20 sec for 40 cycles, final extension 72ºC 10 min). PCR products were analysed on an ABI 3130 Genetic Analyser using an internal size standard (GeneScan 600 LIZ; Applied Biosystems). Microsatellite allele scoring for the Atlantic samples was done following the same criteria and comparing with electropherograms from Mokhtar-Jamaï et al. (2011) on GeneMapper v.3.5 (Applied Biosystems). The different datasets were standardized by reanalysing a subset of the samples from Mokhtar-Jamaï et al. (2011). Appropriate binning of all microsatellite alleles was verified and corrected using the R package "MsatAllele" (Alberto, 2009). Replicate genotyping runs resolved inconsistencies. Genotypes that could not be resolved after replicate runs were omitted from further analyses.

## Note S1.2. Quality check

We used GenClone 2.0 (Arnaud-Haond & Belkhir, 2007) to calculate the number of distinct multi-locus genotypes (MLG) per sample and genotypic richness (Arnaud-Haond et al., 2005). Scoring errors, large allele dropout and null allele frequencies were assessed with MICRO-CHECKER v.2.2.3 (Oosterhout et al., 2004) and we computed the frequencies of null alleles for each locus and sample using FREENA (Chapuis & Estoup, 2007).

## Note S1.3. Population and lineage genetic diversity

Indices of genetic diversity were compared among populations and genetic lineages identified by SODA analysis (see Material and Methods of main manuscript). The total number of alleles per locus, the observed heterozygosity (*H_o_*), gene diversity (*H_e_*; Nei 1973) and the *F_IS_* (Weir & Cockerham, 1984) were computed for each population / lineage with GENETIX 4.05 (Belkhir et al., 2004). We tested linkage equilibrium for each pair of loci over all populations and in each population / lineage using a permutation procedure (n=1000) in GENETIX. For each population / lineage, we tested departure from panmixia using the score tests for heterozygote deficiency implemented in GENEPOP 4.1.4 (Rousset, 2008). Significance of the result was computed by Markov Chain algorithm using default parameters (Guo & Thompson 1992). The allelic richness (*Ar_g_*) and private allelic richness (*Ap_g_*) were estimated for each population / lineage using the rarefaction method (Petit et al. 1998) implemented in ADZE (Szpiech et al., 2008) with *g*, the minimum number of genes at a locus in a population, equals to 18. We compared, *Ar_g_* and *Ap_g_* among the populations and three lineages identified by SODA analysis using a Krukal-Wallis rank sum test followed by pairwise Wilcoxon rank sum tests in R.

# Note S2 – Supplementary results

## Note S2.1. Genetic variability at microsatellite loci

Genotypic richness was close to one in all locations except in the Aegean Sea (AYV, 0.69). Values lower than one could be the result of mishandling during sampling. Considering the low probability of identity (PI = 5*10^-11^), a single representative of each group of similar multi-locus genotypes was kept in the dataset for population-level analyses. No evidence of large allele dropout or scoring errors owing to stutters was found. The estimated frequency of null allele was between 0.01 and 0.1 for Parcla_14 and Parcla_9. All loci were polymorphic with a mean number of 38 alleles per locus (range 16-55 for Parcla_17 and Parcla_9, respectively). Some of the Atlantic populations were almost fixed at one locus (e.g. SEG and LAG are fixed while LAG only harbours 5 alleles for Parcla_12). We find significant linkage disequilibrium between Parcla_12 and Parcla_14 when considering all the populations. Yet, this disequilibrium was not systematically observed at the population level and as such we retained the two loci for the following analyses. At the population level, the estimated frequency of null allele was between 0 for PZO and 0.12 for COR. Significant linkage disequilibrium was observed for 15 different pairs of loci in seven different populations (e.g. Parcla_12 Parcla_17 in CAB; Par-d Parcla_9 in COR).

# Note S3 - Supplementary references

Alberto, F. (2009). MsatAllele_1.0: An R package to visualize the binning of microsatellite alleles. *The Journal of Heredity*, *100*(3), 394–397. <https://doi.org/10.1093/jhered/esn110>

Arnaud-Haond, S., Alberto, F., Teixeira, S., Procaccini, G., Serrão, E. A., & Duarte, C. M. (2005). Assessing genetic diversity in clonal organisms: low diversity or low resolution? Combining power and cost efficiency in selecting markers. *Journal of Heredity*, *96*(4), 434–440. <https://doi.org/10.1093/jhered/esi043>

Arnaud-Haond, S., & Belkhir, K. (2007). genclone: a computer program to analyse genotypic data, test for clonality and describe spatial clonal organization. *Molecular Ecology Notes*, *7*(1), 15–17. <https://doi.org/10.1111/j.1471-8286.2006.01522.x>

Chapuis, M.-P., & Estoup, A. (2007). Microsatellite null alleles and estimation of population differentiation. *Molecular Biology and Evolution*, *24*(3), 621–631. <https://doi.org/10.1093/molbev/msl191>

Guo, S.W., Thompson, E.A. (1992). Performing the exact test of Hardy-Weinberg proportion for multiple alleles. *Biometrics*, 48(2), 361-72. https://doi.org/10.2307/2532296

Mokhtar-Jamaï, K., Pascual, M., Ledoux, J. B., Coma, R., Féral, J. P., Garrabou, J., & Aurelle, D. (2011). From global to local genetic structuring in the red gorgonian *Paramuricea clavata*: the interplay between oceanographic conditions and limited larval dispersal. *Molecular Ecology*, *20*(16), 3291–3305. <https://doi.org/10.1111/j.1365-294x.2011.05176.x>

Oosterhout, C. van, Hutchinson, W. F., Wills, D. P. M., & Shipley, P. (2004). micro-checker: software for identifying and correcting genotyping errors in microsatellite data. *Molecular Ecology Notes*, *4*(3), 535–538. <https://doi.org/10.1111/j.1471-8286.2004.00684.x>

Rousset, F. (2008). genepop’007: a complete re-implementation of the genepop software for Windows and Linux. *Molecular Ecology Resources*, *8*(1), 103–106. <https://doi.org/10.1111/j.1471-8286.2007.01931.x>

Szpiech, Z. A., Jakobsson, M., & Rosenberg, N. A. (2008). ADZE: A rarefaction approach for counting alleles private to combinations of populations. *Bioinformatics*, *24*(21), 2498–2504. <https://doi.org/10.1093/bioinformatics/btn478>

Weir, B. S., & Cockerham, C. C. (1984). Estimating F-statistics for the analysis of population structure. *American Journal of Human Genetics*, *38*(6), 1358–1370. https://doi.org/10.2307/2408641
