# Supplementary material for: Not out of the Mediterranean: Atlantic populations of the gorgonian Paramuricea clavata are a separate sister species under further lineage diversification
Source: Ecol Evol. 2023 Jan 29;13(1):e9740. doi: 10.1002/ece3.9740 (PMC9912747; doi:10.1002/ece3.9740)
Supplement: Supplementary file 3 — Table S1. Table S2. Table S3. Table S4. Table S5. Table S6. [file ECE3-13-e9740-s001.docx]

**Supplemental Information for:**

**Not out of the Mediterranean: Atlantic populations of the gorgonian *Paramuricea* *clavata* are a separate sister species under further diversification**

Márcio A. G. Coelho^1,2*§^, Gareth A. Pearson^1*§^, Joana R. H. Boavida^3^, Diogo Paulo^1^, Didier Aurelle^3,4^, Sophie Arnaud-Haond^5^, Daniel Gómez-Gras^6,7,8^, Nathaniel Bensoussan^3,9^, Paula López-Sendino^9^, Carlo Cerrano^10,11,12,13^, Silvija Kipson^14,15^, Tatjana Bakran-Petricioli^14^, Eliana Ferretti^16,17^, Cristina Linares^7,8^, Joaquim Garrabou^3,9^, Ester A. Serrão^1,18^, Jean-Baptiste Ledoux^19*^

Table S1 - Location, coordinates, depth and sample size of samples collected for the study. Correspondence with the genetic cluster / lineages identified by STRUCTURE / SODA analysis, as shown in colours in the figures of the main manuscript are as follows: 1 – Atlantic purple morphotype of Paramuricea cf. grayi; 2 - Atlantic yellow morphotype of Paramuricea cf. grayi; 3 – Mediterranean P. clavata. The asterisk (*) denotes new locations relative to the dataset of Mokhtar-Jamaï et al. (2011). Values in parenthesis within the sample size column represent the number of samples analysed after the filtration steps (microsatellite dataset). The last three entries highlighted in grey refer to samples of Eunicella verrucosa used to assemble the reference transcriptome used in the reciprocal blast to identify additional putative single-copy orthologues (BioSamples: SAMN28899329-SAMN28899331; see Material and Methods).

| Dataset | Region/genetic cluster | Location name | Location code | Latitude | Longitude | Depth (m) | Sample size |
| --- | --- | --- | --- | --- | --- | --- | --- |
| Microsat | NE Atlantic/1 | Farilhão da Cova (Berlengas, West Iberia) * | FAR | 39.476 | -9.546 | 10 | 27 (27) |
| Microsat | NE Atlantic/1 | Rabo d'Asno (Berlengas, West Iberia) * | RAB | 39.558 | -9.752 | 26 | 39 (39) |
| Microsat | NE Atlantic/1 | Pedra do Coral (Cape Espichel, West Iberia) * | COR | 38.419 | -9.257 | 55 | 45 (45) |
| Microsat | NE Atlantic/1 | Catrapona (Cape Espichel, West Iberia) * | CAT | 38.419 | -9.253 | 40 | 84 (73) |
| Microsat | NE Atlantic/1 | Baixa do CPAS (Sines, West Iberia) * | SIN | 37.898 | -8.923 | 60 | 36 (32) |
| Microsat | NE Atlantic/2 | Tunel do Segredo (Sagres, Southwest Iberia) * | SEG | 37.006 | -8.927 | 15 | 41 (30) |
| Microsat | NE Atlantic/2 | Baleeira (Sagres, Southwest Iberia) * | BAL | 37.009 | -8.925 | 22 | 12 (10) |
| Microsat | NE Atlantic/2 | Pedra dos 60m (Lagos, Southwest Iberia) * | LAG | 37.004 | -8.704 | 60 | 34 (34) |
| Microsat | NE Atlantic/2 | Pedra do Barril (Tavira, Southwest Iberia) * | TAV | 37.057 | -7.635 | 30 | 36 (34) |
| Microsat | Mediterranean Sea/3 | Monte-Hacho (Ceuta, Alboran Sea, Spain) | MTH | 35.918 | -5.300 | 31 | 54 (25) |
| Microsat | Mediterranean Sea/3 | Marinasmir (Alboran Sea, Morocco) | MIR | 35.752 | -5.313 | 40 | 14 (11) |
| Microsat | Mediterranean Sea/3 | Martyl (Alboran Sea, Marocco) | TYL | 35.642 | -5.248 | 35 | 28 (14) |
| Microsat | Mediterranean Sea/3 | Bajo de Fuera (Cap de Palos, Spain) | CPS | 37.652 | -0.652 | 40 | 34 (28) |
| Microsat | Mediterranean Sea/3 | Bledes (Na Gorra) (Ibiza Island, Spain) | IBZ | 38.970 | 1.670 | 37 | 31 (30) |
| Microsat | Mediterranean Sea/3 | L’Imperial (Cabrera Island, Spain) | CAB | 39.124 | 2.960 | 40 | 19 (12) |
| Microsat | Mediterranean Sea/3 | Bajo del Carallot (Columbretes Island, Spain) | CLB | 39.892 | 0.670 | 40 | 33 (14) |
| Microsat | Mediterranean Sea/3 | Carall Bernat (Medes Islands, Spain) | MES | 42.052 | 3.222 | 20 | 32 (22) |
| Microsat | Mediterranean Sea/3 | Cova de la Vaca (Medes Islands, Spain) | CDV | 42.050 | 3.225 | 17 | 46 (32) |
| Microsat | Mediterranean Sea/3 | Medan gras, Pota del llop (Medes, Spain) | PLL | 42.050 | 3.225 | 35 | 29 (27) |
| Microsat | Mediterranean Sea/3 | Pota del Llop (Medes Islands, Spain) | MED | 42.048 | 3.226 | 15 | 28 (25) |
| Microsat | Mediterranean Sea/3 | Medallot (Medes Islands, Spain) | MET | 42.042 | 3.228 | 20 | 30 (14) |
| Microsat | Mediterranean Sea/3 | Cova de la Vaca (Medes Islands, Spain)* | VAC | 42.048 | 3.226 | 20 | 29 (23) |
| Microsat | Mediterranean Sea/3 | Punta Falconera (Cap de Creus, Spain) | CCS | 42.232 | 3.219 | 25 | 30 (26) |
| Microsat | Mediterranean Sea/3 | Sec Rederis (Banyuls, France) | SRE | 42.464 | 3.167 | 23 | 37 (10) |
| Microsat | Mediterranean Sea/3 | Pharillons (île Maïre, Marseille, France) | PHL | 43.207 | 5.338 | 40 | 31 (28) |
| Microsat | Mediterranean Sea/3 | Riou Sud (Marseille, France) | RIO | 43.173 | 5.390 | 25 | 35 (31) |
| Microsat | Mediterranean Sea/3 | Riou Sud (Marseille, France) | RIS | 43.173 | 5.390 | 40 | 33 (25) |
| Microsat | Mediterranean Sea/3 | Morgiou (Marseille, France) | MOR | 43.173 | 5.393 | 30 | 35 (30_ |
| Microsat | Mediterranean Sea/3 | Petit Congloue (France) | PCL | 43.179 | 5.396 | 20 | 38 (17) |
| Microsat | Mediterranean Sea/3 | Petit Congloue (France) | PTC | 43.179 | 5.396 | 10 | 33 (32) |
| Microsat | Mediterranean Sea/3 | Castelvieil (Marseille, France) | CAS | 43.176 | 5.402 | 10 | 33 (30) |
| Microsat | Mediterranean Sea/3 | Grand Congloue (Marseille, France) | GCL | 43.201 | 5.451 | 7 | 42 (35) |
| Microsat | Mediterranean Sea/3 | Grotte Peres (Marseille, France) | GPR | 43.198 | 5.499 | 20 | 39 (38) |
| Microsat | Mediterranean Sea/3 | Montrémian (Port-Cros, France) | MTM | 43.019 | 6.363 | 25 | 30 (28) |
| Microsat | Mediterranean Sea/3 | Pointe Vaisseau (Port-Cros, France) | PTV | 42.995 | 6.407 | 25 | 29 (29) |
| Microsat | Mediterranean Sea/3 | Imperiales de Terre (Marseille, France) | IMP | 43.173 | 5.393 | 8 | 41 (33) |
| Microsat | Mediterranean Sea/3 | Gabiniere (Port-Cros, France) | GAB | 42.989 | 6.397 | 22 | 32 (32) |
| Microsat | Mediterranean Sea/3 | Palazzu (North Corsica, France)* | PZU | 42.380 | 8.546 | 24 | 65 (53) |
| Microsat | Mediterranean Sea/3 | Palazzu (North Corsica, France) | PLU | 42.380 | 8.546 | 28 | 32 (15) |
| Microsat | Mediterranean Sea/3 | Palazzinu (North Corsica, France) | PZO | 42.380 | 8.549 | 25 | 41 (16) |
| Microsat | Mediterranean Sea/3 | Punta Muchillina (North Corsica, France) | PML | 42.332 | 8.553 | 20 | 32 (23) |
| Microsat | Mediterranean Sea/3 | Garganellu (North Corsica, France) | GGL | 42.373 | 8.537 | 20 | 31 (10) |
| Microsat | Mediterranean Sea/3 | Altare (Portofino, Italy) | ALT | 44.306 | 9.196 | 25 | 31 (25) |
| Microsat | Mediterranean Sea/3 | Ischia (Italy) | SLO | 40.692 | 13.894 | 35 | 32 (11) |
| Microsat | Mediterranean Sea/3 | Fulija Island (Dugi Otok, Adriatic Sea, Croatia) | FUL | 44.018 | 15.111 | 40 | 33 (31) |
| Microsat | Mediterranean Sea/3 | Ayvalik (Ezerbey Sigiligi, Aegean Sea, Turkey) | AYV | 39.370 | 26.577 | 45 | 21 (14) |
| RNA-seq | Mediterranean Sea/3 | La Vaca (Medes, Catalonia, Spain) | VAC | 42.048 | 3.226 | 18 - 20 | 8 |
| RNA-seq | Mediterranean Sea/3 | Altare (Portofino, Italy) | ALT | 44.309 | 9.179 | 35 - 37 | 6 |
| RNA-seq | Mediterranean Sea/3 | Balun (Kornati, Croatia) | BALU | 43.805 | 15.255 | 33 - 36 | 7 |
| RNA-seq | NE Atlantic/2 | Baleeira (Sagres, Southwest Iberia) | BAL | 37.012 | -8.924 | 15 | 6 |
| RNA-seq | NE Atlantic/1 & 2 | P39 (Cape Espichel, West Iberia) | P39 | 38.412 | -9.241 | 31-35 | 3 |
| RNA-seq | NE Atlantic/2 | Pedra do Barril (Tavira, Southwest Iberia) | TAV | 37.057 | -7.635 | 26.4 | 1 |
| RNA-seq | NE Atlantic | Professor Luiz Saldanha Marine Park (Arrabida, West Iberia) | PMPLS | - | - | - | 1 |
| RNA-seq | NE Atlantic | P39 (Cape Espichel, West Iberia) | P39 | 38.412 | -9.241 | 31-35 | 1 |
| RNA-seq | NE Atlantic | Sagres (Southwest Iberia) | SAG | 36.984 | - 9.046 | 158 | 1 |

Table S2 - Summary statistics of the RNA-seq dataset of *Paramuricea* cf. *grayi* (Atlantic) and *P. clavata* (Mediterranean) used for phylogenomic analysis. Transcriptome assemblies were performed on the filtered dataset for samples from each population studied. Note that the final transcriptomes were subjected to additional quality-control filtering and curating steps (see Material and Methods). Sample of *P.* cf. *grayi* from Baleeira, Tavira and 19-0046 from P39 were from the yellow lineage, whereas the remainder from P39 (19-0054 and 19-0057) belonged to the purple lineage. Entries with two BioSample accession numbers refer to pooled sequence data from the same specimen sampled at two distinct time points during a heat-stress experiment conducted by Coelho et al. (*in prep*).

| **Species** | **Population** | **Sample ID** | **Number raw reads (1+2; M)** | **Number filtered reads (1+2; M)** | **BioSample Accessions** |
| --- | --- | --- | --- | --- | --- |
| *Paramuricea clavata* | Balun (Croatia) | BALU3 | 71.35 | 66.78 | SAMN28899278 & SAMN28899281 |
| *Paramuricea clavata* | Balun (Croatia) | BALU4 | 80.68 | 76.49 | SAMN28899279 & SAMN28899282 |
| *Paramuricea clavata* | Balun (Croatia) | BALU7 | 81.67 | 70.05 | SAMN28899280 & SAMN28899283 |
| *Paramuricea clavata* | Balun (Croatia) | BALU1 | 74.27 | 61.69 | SAMN28899284 & SAMN28899287 |
| *Paramuricea clavata* | Balun (Croatia) | BALU5 | 39.02 | 38.63 | SAMN28899285 |
| *Paramuricea clavata* | Balun (Croatia) | BALU6 | 71.70 | 49.01 | SAMN28899286 & SAMN28899288 |
| *Paramuricea clavata* | Balun (Croatia) | BALU9 | 35.30 | 27.33 | SAMN28899289 |
| *Paramuricea clavata* | Altare (Italy) | ALT4 | 56.11 | 47.18 | SAMN28899290 & SAMN28899294 |
| *Paramuricea clavata* | Altare (Italy) | ALT7 | 59.47 | 52.89 | SAMN28899291 & SAMN28899295 |
| *Paramuricea clavata* | Altare (Italy) | ALT8 | 59.83 | 46.42 | SAMN28899292 & SAMN28899296 |
| *Paramuricea clavata* | Altare (Italy) | ALT3 | 32.41 | 24.38 | SAMN28899293 |
| *Paramuricea clavata* | Altare (Italy) | ALT1 | 59.17 | 49.86 | SAMN28899297 & SAMN28899299 |
| *Paramuricea clavata* | Altare (Italy) | ALT6 | 68.73 | 46.53 | SAMN28899298 & SAMN28899300 |
| *Paramuricea* cf. *grayi* | Baleeira (Portugal) | BAL3 | 60.47 | 46.64 | SAMN28899305 & SAMN28899308 |
| *Paramuricea* cf. *grayi* | Baleeira (Portugal) | BAL4 | 35.02 | 26.23 | SAMN28899306 |
| *Paramuricea* cf. *grayi* | Baleeira (Portugal) | BAL8 | 70.96 | 50.56 | SAMN28899307 & SAMN28899309 |
| *Paramuricea* cf. *grayi* | Baleeira (Portugal) | BAL1 | 57.19 | 43.45 | SAMN28899310 & SAMN28899313 |
| *Paramuricea* cf. *grayi* | Baleeira (Portugal) | BAL5 | 70.85 | 58.83 | SAMN28899311 & SAMN28899314 |
| *Paramuricea* cf. *grayi* | Baleeira (Portugal) | BAL6 | 53.36 | 46.35 | SAMN28899312 & SAMN28899315 |
| *Paramuricea clavata* | La Vaca (Spain) | VAC3 | 76.51 | 61.63 | SAMN28899316 & SAMN28899320 |
| *Paramuricea clavata* | La Vaca (Spain) | VAC4 | 78.19 | 65.83 | SAMN28899317 & SAMN28899321 |
| *Paramuricea clavata* | La Vaca (Spain) | VAC7 | 82.54 | 75.50 | SAMN28899318 & SAMN28899322 |
| *Paramuricea clavata* | La Vaca (Spain) | VAC8 | 33.19 | 19.57 | SAMN28899319 |
| *Paramuricea clavata* | La Vaca (Spain) | VAC1 | 64.22 | 45.51 | SAMN28899323 & SAMN28899326 |
| *Paramuricea clavata* | La Vaca (Spain) | VAC5 | 37.24 | 24.22 | SAMN28899324 |
| *Paramuricea clavata* | La Vaca (Spain) | VAC6 | 76.22 | 67.95 | SAMN28899325 & SAMN28899327 |
| *Paramuricea clavata* | La Vaca (Spain) | VAC9 | 42.83 | 33.05 | SAMN28899328 |
| *Paramuricea* cf. *grayi* | P39 (Portugal) | 19-0054 | 33.41 | 32.77 | SAMN28899301 |
| *Paramuricea* cf. *grayi* | P39 (Portugal) | 19-0057 | 27.47 | 26.73 | SAMN28899302 |
| *Paramuricea* cf. *grayi* | P39 (Portugal) | 19-0046 | 28.00 | 18.76 | SAMN28899303 |
| *Paramuricea* cf. *grayi* | Tavira (Portugal) | 19-0097 | 32.73 | 29.00 | SAMN28899304 |

Table S3 - Summary statistics assessing the quality and completeness of the transcriptomes assembled *de novo* with rnaSPAdes on the *Paramuricea* cf. *grayi* (Atlantic) and *P. clavata* (Mediterranean) RNA-seq data. We compared a total of four independent transcriptome assemblies reconstructed with samples from each population studied by Gómez-Gras et al. (*accepted*): one for *P.* cf. *grayi* using all the sequence data from Baleeira (Sagres, Portugal) and three for *P. clavata* using the sequence data from Altare (Italy), La Vaca (Spain) or Balun (Croatia). Transcriptome assemblies were evaluated with Transrate in read-metrics mode and BUSCO reports. For more information on the quality metrics reported by Transrate refer to the Project webpage https://hibberdlab.com/transrate/metrics.html. The BUSCO notation for the genes queried are: C: complete [S: single-copy, D: duplicated], F: fragmented and M: missing.

| **Evaluation tool** | **Quality metric** | ***P.* cf. *grayi* (Baleeira)** | ***P. clavata* (Altare)** | ***P. clavata* (La Vaca)** | ***P. clavata* (Balun)** |
| --- | --- | --- | --- | --- | --- |
| Transrate | n_seqs | 524051 | 511091 | 681261 | 548902 |
| Transrate | smallest | 49 | 49 | 49 | 49 |
| Transrate | largest | 48484 | 39477 | 42114 | 29928 |
| Transrate | n_bases | 351727338 | 355175805 | 489821096 | 391519195 |
| Transrate | mean_len | 618.9338 | 646.4183 | 674.4854 | 666.4069 |
| Transrate | n_under_200 | 157612 | 142963 | 174553 | 148051 |
| Transrate | n_over_1k | 89120 | 96483 | 133602 | 106380 |
| Transrate | n_over_10k | 444 | 392 | 534 | 493 |
| Transrate | n_with_orf | 85181 | 85908 | 111171 | 92721 |
| Transrate | mean_orf_percent | 51.4718 | 50.1831 | 47.3633 | 49.0292 |
| Transrate | n90 | 312 | 335 | 341 | 339 |
| Transrate | n70 | 995 | 958 | 947 | 976 |
| Transrate | n50 | 2068 | 1830 | 1803 | 1866 |
| Transrate | n30 | 3581 | 3178 | 3164 | 3240 |
| Transrate | n10 | 9112 | 7700 | 7296 | 7714 |
| Transrate | gc | 0.4122 | 0.4020 | 0.4033 | 0.4047 |
| Transrate | gc_skew | 0.0024 | 0.0029 | 0.0030 | 0.0032 |
| Transrate | at_skew | -0.0086 | -0.0079 | -0.0082 | -0.0069 |
| Transrate | cpg_ratio | 1.9550 | 1.9473 | 1.9665 | 1.9620 |
| Transrate | bases_n | 94900 | 157200 | 214060 | 165955 |
| Transrate | proportion_n | 0.0003 | 0.0004 | 0.0004 | 0.0004 |
| Transrate | linguistic_complexity | 0.1168 | 0.1222 | 0.1254 | 0.1244 |
| Transrate | fragments | 136031945 | 133629886 | 10754738 | 16698608 |
| Transrate | fragments_mapped | 132548231 | 122799157 | 8806745 | 16412716 |
| Transrate | p_fragments_mapped | 0.9744 | 0.9190 | 0.8189 | 0.9829 |
| Transrate | good_mappings | 123407614 | 107320353 | 6499989 | 15544912 |
| Transrate | p_good_mapping | 0.9072 | 0.8031 | 0.6044 | 0.9309 |
| Transrate | bad_mappings | 9140617 | 15478804 | 2306756 | 867804 |
| Transrate | potential_bridges | 98545 | 123348 | 15187 | 22258 |
| Transrate | bases_uncovered | 17975940 | 15322771 | 303841235 | 229923640 |
| Transrate | p_bases_uncovered | 0.0511 | 0.0431 | 0.6203 | 0.5873 |
| Transrate | contigs_uncovbase | 154037 | 186502 | 675165 | 545324 |
| Transrate | p_contigs_uncovbase | 0.2939 | 0.3649 | 0.9911 | 0.9935 |
| Transrate | contigs_uncovered | 2320 | 2182 | 394336 | 356159 |
| Transrate | p_contigs_uncovered | 0.0044 | 0.0043 | 0.5788 | 0.6489 |
| Transrate | contigs_lowcovered | 2653 | 2979 | 503385 | 411398 |
| Transrate | p_contigs_lowcovered | 0.0051 | 0.0058 | 0.7389 | 0.7495 |
| Transrate | contigs_segmented | 43868 | 43693 | 5651 | 8666 |
| Transrate | p_contigs_segmented | 0.0837 | 0.0855 | 0.0083 | 0.0158 |
| Transrate | score | 0.3556 | 0.3288 | 0.018 | 0.025 |
| Transrate | optimal_score | 0.4864 | 0.4318 | 0.2599 | 0.4268 |
| Transrate | cutoff | 0.0306 | 0.0330 | 0.3393 | 0.3967 |
| BUSCO | Total number of core genes queried | 978 | 978 | 978 | 978 |
| BUSCO | Complete | 958 (97.96%) | 953 (97.44%) | 874 (89.37%) | 905 (92.54%) |
| BUSCO | Complete + Partial | 965 (98.67%) | 965 (98.67%) | 904 (92.43%) | 933 (95.40%) |
| BUSCO | Number of missing core genes | 13 (1.33%) | 13 (1.33%) | 74 (7.57%) | 45 (4.60%) |
| BUSCO | Average number of orthologs per core genes | 2 | 1.78 | 1.17 | 1.27 |
| BUSCO | % of detected core genes that have more than 1 ortholog | 68.06 | 56.56 | 15.56 | 23.31 |
| BUSCO | Scores in BUSCO format | C:98.0%[S:31.3%,D:66.7%],F:0.7%,M:1.3% | C:97.4%[S:42.3%,D:55.1%],F:1.2%,M:1.4% | C:89.4%[S:75.5%,D:13.9%],F:3.1%,M:7.5% | C:92.6%[S:71.0%,D:21.6%],F:2.9%,M:4.5% |

Table S4 – Genetic diversity of each sample. r: frequency of null alleles estimated in FREENA; H_o_: observed heterozygosity; H_e_: gene diversity (Nei 1973); F_IS_: Weir & Cockerham (1984) estimator of F_IS_ (bold values are significant at 0.01); Ar_(18)_ and Ap_(18)_ rarefied allelic and private allelic richness considering a minimum of 18 genes at a locus in a sample.

|  |  | r | H_o_ | H_e_ | F_IS_ | Ar_(18)_ | Ap_(18)_ |
| --- | --- | --- | --- | --- | --- | --- | --- |
| MEDITERRANEAN | AYV | 0.02 | 0.62 | 0.57 | -0.084 | 4.79 | 0.17 |
|  | FUL | 0.03 | 0.63 | 0.66 | 0.045 | 4.53 | 0.04 |
|  | SLO | 0.04 | 0.68 | 0.76 | 0.104 | 6.75 | 0.14 |
|  | ALT | 0.01 | 0.7 | 0.73 | 0.036 | 6.51 | 0.07 |
|  | GGL | 0.03 | 0.73 | 0.77 | 0.051 | 7.17 | 0 |
|  | PML | 0.02 | 0.69 | 0.72 | 0.042 | 5.91 | 0 |
|  | PZO | 0 | 0.8 | 0.77 | -0.043 | 7.31 | 0.06 |
|  | PLU | 0.03 | 0.76 | 0.78 | 0.031 | 6.63 | 0 |
|  | PZU | 0.01 | 0.77 | 0.76 | -0.012 | 7.08 | 0 |
|  | GAB | 0.03 | 0.7 | 0.76 | 0.07 | 7.59 | 0.02 |
|  | IMP | 0.04 | 0.71 | 0.75 | **0.062** | 7.87 | 0.11 |
|  | PTV | 0.02 | 0.66 | 0.67 | 0.029 | 6.77 | 0 |
|  | MTM | 0.04 | 0.63 | 0.67 | 0.075 | 5.89 | 0.06 |
|  | GPR | 0.01 | 0.76 | 0.77 | 0.016 | 7.82 | 0.14 |
|  | GCL | 0.03 | 0.77 | 0.8 | 0.031 | 7.17 | 0 |
|  | CAS | 0.05 | 0.61 | 0.71 | **0.144** | 6.64 | 0 |
|  | PTC | 0.04 | 0.72 | 0.8 | **0.093** | 7.74 | 0.01 |
|  | PCL | 0.03 | 0.7 | 0.75 | 0.07 | 7.68 | 0.01 |
|  | MOR | 0.03 | 0.71 | 0.76 | 0.066 | 7.65 | 0 |
|  | RIS | 0.04 | 0.69 | 0.75 | **0.084** | 7.49 | 0 |
|  | RIO | 0.06 | 0.65 | 0.76 | **0.156** | 7.91 | 0.01 |
|  | PHL | 0.03 | 0.75 | 0.81 | 0.07 | 7.92 | 0.12 |
|  | SRE | 0.07 | 0.55 | 0.63 | 0.137 | 6.17 | 0 |
|  | CCS | 0.04 | 0.69 | 0.75 | 0.077 | 6.86 | 0 |
|  | VAC | 0 | 0.75 | 0.75 | 0 | 6.87 | 0.03 |
|  | MET | 0.07 | 0.6 | 0.72 | **0.179** | 7.3 | 0.01 |
|  | MED | 0.01 | 0.75 | 0.78 | 0.038 | 7.52 | 0.07 |
|  | PLL | 0.01 | 0.71 | 0.75 | 0.049 | 7.82 | 0.11 |
|  | CDV | 0.03 | 0.68 | 0.74 | 0.074 | 7.02 | 0.06 |
|  | MES | 0.01 | 0.71 | 0.73 | 0.023 | 7.05 | 0 |
|  | CLB | 0.04 | 0.68 | 0.78 | 0.132 | 6.54 | 0 |
|  | CAB | 0.01 | 0.67 | 0.64 | -0.039 | 4.98 | 0.25 |
|  | IBZ | 0.05 | 0.65 | 0.66 | **0.015** | 5.68 | 0.09 |
|  | CPS | 0.08 | 0.62 | 0.73 | **0.15** | 5.79 | 0 |
|  | TYL | 0.09 | 0.56 | 0.76 | **0.268** | 6.62 | 0.36 |
|  | MIR | 0.08 | 0.64 | 0.74 | **0.145** | 6.07 | 0.16 |
|  | MTH | 0.1 | 0.61 | 0.77 | **0.21** | 6.53 | 0.08 |
| ATLANTIC | TAV | 0.1 | 0.44 | 0.62 | **0.304** | 5.99 | 1.52 |
|  | LAG | 0.01 | 0.58 | 0.58 | -0.003 | 4.53 | 0.64 |
|  | BAL | 0.02 | 0.57 | 0.5 | -0.133 | 3.5 | 0.56 |
|  | SEG | 0.05 | 0.54 | 0.56 | 0.04 | 4.24 | 0.59 |
|  | SIN | 0.09 | 0.61 | 0.72 | **0.152** | 6.11 | 0.93 |
|  | CAT | 0.07 | 0.61 | 0.69 | **0.115** | 6.02 | 0.36 |
|  | COR | 0.12 | 0.56 | 0.77 | **0.285** | 6.66 | 0.22 |
|  | RAB | 0.05 | 0.74 | 0.78 | 0.043 | 6.21 | 0.5 |
|  | FAR | 0.04 | 0.58 | 0.62 | 0.069 | 4.57 | 0.31 |

Table S5 – Results of the Analysis of Molecular Variance and related F-statistics. Populations are grouped according to genetic cluster / lineages identified by STRUCTURE / SODA analysis (d.f.=degree of freedom; Est. Var.=Estimated variability and % = percentage of variability explained by the considered grouping). The significance of the F-statistics was tested using a permutation procedure (n=1000).

| Source | d.f. | Est. Var. | % |  | F-statistics | Value | P(rand >= data) |
| --- | --- | --- | --- | --- | --- | --- | --- |
| Among Lineages | 2 | 0.385 | 14% |  | F_rt_ | 0.136 | 0.001 |
| Among Populations | 43 | 0.283 | 10% |  | F_sr_ | 0.115 | 0.001 |
| Among Individuals | 1177 | 0.178 | 6% |  | F_st_ | 0.236 | 0.001 |
| Within Individual | 1223 | 1.991 | 70% |  | F_is_ | 0.082 | 0.001 |
| Total | 2445 | 2.838 | 100% |  | F_it_ | 0.298 | 0.001 |


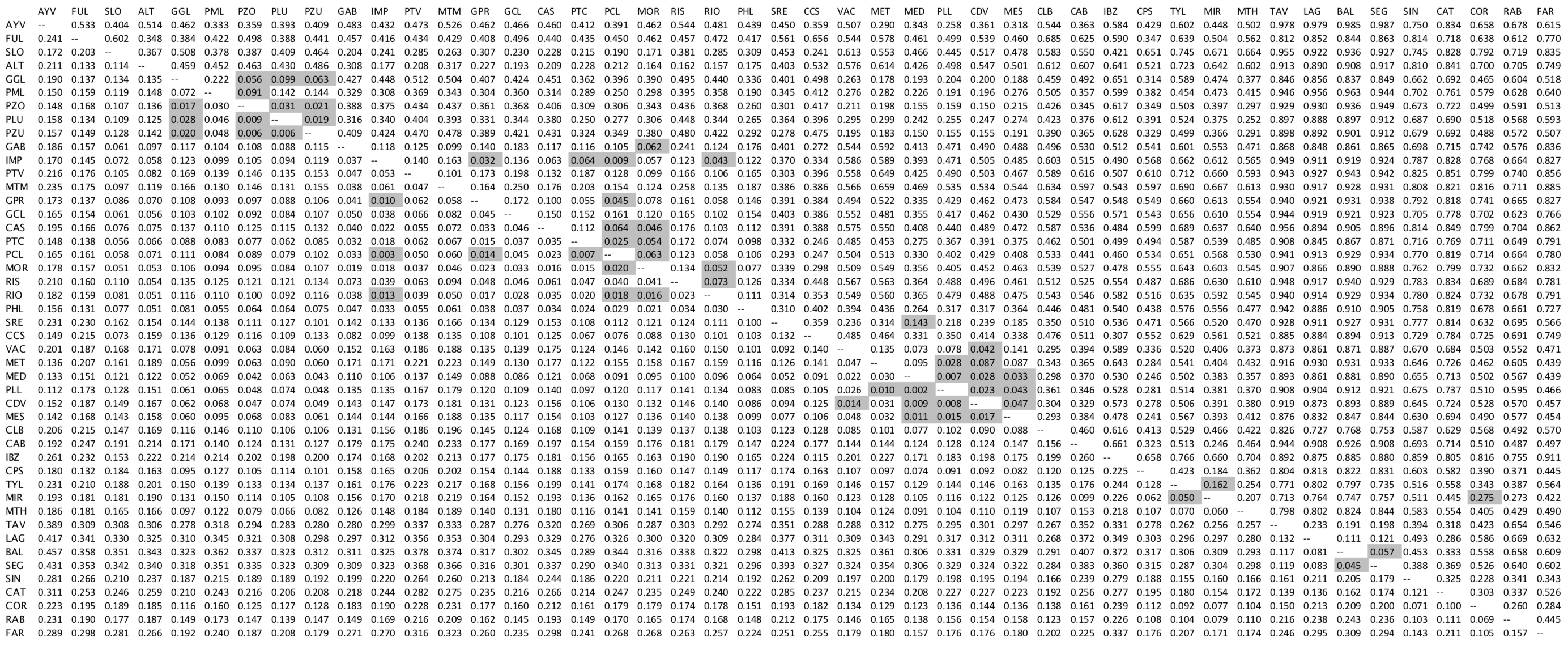
Table S6 – Pairwise G_ST_ (below) and D_EST_ (above) the diagonal. All values but the shaded ones are significant at 0.001 after FDR correction. Location codes follow those used in Table S1 and Figure
